# A streamlined CRISPR-based test for tuberculosis detection directly from sputum

**DOI:** 10.1126/sciadv.adx2067

**Published:** 2025-08-06

**Authors:** Alexandra G. Bell, Owen R. S. Dunkley, Nisha H. Modi, Yujia Huang, Soleil Tseng, Robert Reiss, Naranjargal Daivaa, J. Lucian Davis, Deninson Alejandro Vargas, Padmapriya Banada, Yingda L. Xie, Cameron Myhrvold

**Affiliations:** ^1^ Department of Molecular Biology, Princeton University, Princeton NJ 08544, USA.; ^2^ Public Health Research Institute, Department of Medicine, Rutgers New Jersey Medical School, Newark, NJ 07103, USA.; ^3^ Department of Epidemiology of Microbial Diseases, Yale School of Public Health, New Haven, CT 06510, USA.; ^4^ Pulmonary, Critical Care, and Sleep Medicine Section, Yale School of Medicine, New Haven, CT 06520, USA.; ^5^ Centro Internacional de Entrenamiento e Investigaciones Médicas (CIDEIM), Cali, Colombia.; ^6^ Universidad Icesi, Cali, Colombia.; ^7^ Department of Chemical and Biological Engineering, Princeton University, Princeton, NJ 08544, USA.; ^8^ Omenn-Darling Bioengineering Institute, Princeton University, Princeton, NJ 08544, USA.; ^9^ Department of Chemistry, Princeton University, Princeton, NJ 08544, USA.

## Abstract

*Mycobacterium tuberculosis* (*Mtb*) is a major threat to global health, and there is an urgent need for affordable, simple tuberculosis (TB) diagnosis in underresourced areas. Here, we combine recombinase polymerase amplification with Cas13a and Cas12a detection to create two parallelized one-pot assays that detect two conserved elements of *Mtb* (*IS6110* and *IS1081*) and a human DNA internal control. These assays are compatible with lateral flow and can be readily lyophilized. Our final assay showed a limit of detection of 69.0 CFU per milliliter for *Mtb* H37Rv and 80.5 CFU per milliliter for *Mycobacterium bovis* BCG in spiked sputum, with no cross-reactivity to diverse bacterial or fungal isolates. Clinical tests on 13 blinded sputum samples revealed 100% (six of six) sensitivity and 100% (seven of seven) specificity compared to culture. SHINE-TB streamlines TB diagnosis from sample to answer by combining amplification and detection while being compatible with lateral flow and lyophilization.

## Teaser

A streamlined, CRISPR-based method is developed for detecting tuberculosis directly from sputum samples.

## INTRODUCTION

Tuberculosis (TB), caused by the pathogen *Mycobacterium tuberculosis* (*Mtb*), is a major threat to global health and is difficult to diagnose (*1*). Current TB diagnostic methods are limited by trade-offs among sensitivity, turnaround time, and accessibility. Culture-based methods are very sensitive, but the turnaround time is slow (weeks) (*2*). Conversely, sputum smear microscopy is rapid (~1-hour turnaround) but has limited sensitivity and specificity, in addition to relying on user expertise (*3*–*5*). Polymerase chain reaction (PCR)–based tests, such as Xpert^®^ MTB/RIF (for TB and rifampicin resistance detection), are sensitive, but they require specialized equipment with an integrated thermocycler and proprietary single-use cartridges (*6*–*8*). Thus, there remains a critical unmet need for sensitive point-of-care TB diagnostics in low-resourced areas.

CRISPR-based diagnostics (CRISPR-Dx) have the potential to bridge the accessibility and accuracy gap by leveraging the high specificity and collateral cleavage activities of CRISPR-associated proteins such as Cas12 and Cas13 and pairing them with isothermal amplification techniques (*9*–*16*). Upon target recognition, Cas12 and Cas13 cleave their targets in cis and cleave nearby single-stranded DNA or RNA, respectively, in trans (*12*, *13*, *17*). Thus, by including a quenched fluorescent reporter with a cleavable nucleic acid linker, target recognition can be converted to a fluorescent signal (*9*). Cas12 and Cas13 require a high-degree of CRISPR RNA (crRNA)–target complementarity to activate their nuclease domains, making reporter cleavage highly specific to the presence of a target sequence (*12*, *17*, *18*). Two representative subtypes, Cas12a and Cas13a, both exhibit high turnover catalytic activity, and pairing them with an excess of cleavable nucleic acid reporter provides improved signal amplification (*9*, *10*, *12*, *19*–*21*). This makes them well suited for integration with isothermal amplification, which has been frequently used for TB detection (*22*–*26*). When combined with isothermal amplification, these properties enable robust, highly specific detection with clinically relevant sensitivity, without the need for thermocycling (*10*–*12*, *27*).

Several groups have used CRISPR-Dx paired with isothermal amplification or PCR to address accessibility issues in TB diagnosis (table S1) (*28*–*37*). However, these methods require user manipulations and specialized instrumentation between the preamplification of the sample and detection, making these assays hard to deploy and exposing the workflow to potential sources of contamination. Peng and colleagues developed a Cas12-based format that combines amplification and detection but requires an automated nucleic acid extractor for sputum samples and a real-time PCR instrument for readout for both sputum and cell-free samples (*27*, *38*). Therefore, there is an unmet need for CRISPR-Dx methods designed to work in constrained environments with simplified sample preparation techniques for the detection of TB-causing mycobacteria without specialized instrumentation.

In this study, we have developed SHINE-TB from our general-purpose diagnostic platform, SHINE (Streamlined Highlighting of Infections to Navigate Epidemics) (*14*, *16*). SHINE-TB consists of two parallelized one-pot reactions that combine isothermal amplification by recombinase polymerase amplification (RPA) (*39*), in vitro transcription, and detection, including a Cas13a assay for detecting two conserved elements in the *Mtb* genome and a Cas12a assay for detecting human DNA as an internal control. Combined with an efficient sample processing method and an optimized CRISPR Cas13a/Cas12a assay, we present a simple, point-of-care–oriented, rapid, and highly sensitive TB detection system that requires limited low-cost instrumentation (fig. S1) (*40*).

## RESULTS

### One-pot Cas12a assay accurately detects *Mtb* at genome-level sensitivity

**Fig. 1.**
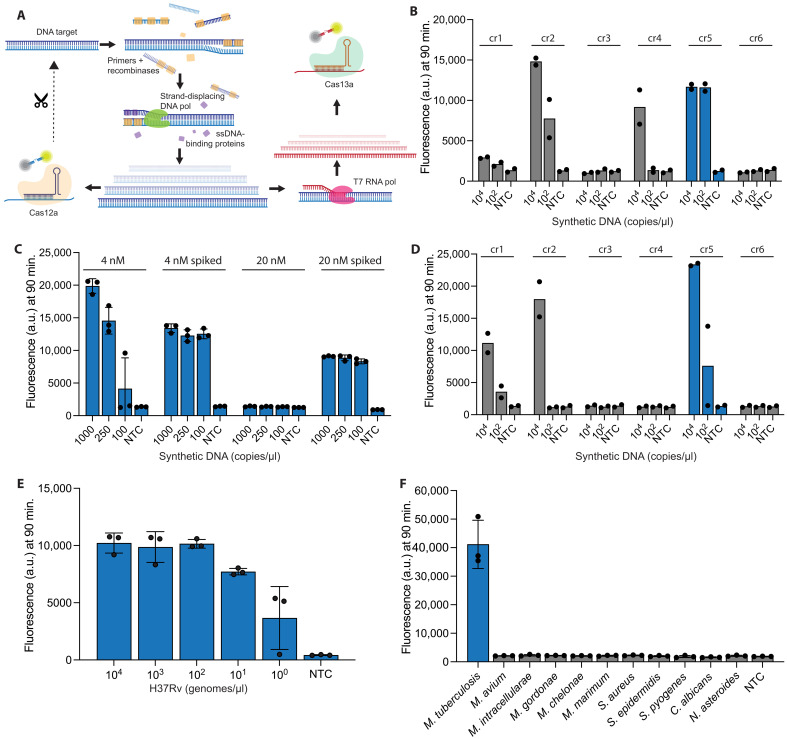
CRISPR-Cas12a assay optimization for detecting *Mtb*. (**A**) Schematic of Cas-12a/Cas13a-based CRISPR reaction, where DNA is amplified using target specific primers, recombinases, strand-displacing polymerase, and single-stranded DNA (ssDNA)–binding proteins and directly detected by Cas12a or transcribed to RNA with T7 reverse transcription for detection with Cas13a. (**B**) Cas12a detecting synthetic DNA for six different crRNA-primer designs. Amplification was allowed to proceed for 30 min before the addition of crRNA and Cas12a. (**C**) Cas12a detecting synthetic DNA with varying concentrations of Cas12a. Molar concentration alone indicates all components present from the start of the reaction. Molar concentration spiked indicates amplification was allowed to proceed for 30 min before the addition of crRNA and Cas12a. (**D**) Cas12a detecting synthetic DNA in a one-pot format, as all components are present from the start of the reaction. (**E**) Performance of Cas12a assay for decreasing concentrations of H37Rv genome. All bars are significant with *P*_adj_ < 0.0001 except for 10^0^ that has *P* = 0.0410. (**F**) Specificity panel for Cas12a assay against various NTMs and other bacteria/fungi at 1 ng/μl. All bars except for *M. tuberculosis* are not significant (n.s.). In (B) and (D), error bars: SD based on *n* = 2 technical replicates. In (C), (E), and (F), error bars: SD based on *n* = 3 technical replicates. In (E) and (F), one-way analysis of variance (ANOVA) with Dunnett’s test was performed, and *P*_adj_ values were calculated compared to the nontarget control (NTC) that contained water. a.u., arbitrary units.

We first explored a streamlined Cas12a-based diagnostic workflow for *Mtb*, where isothermal amplification of the DNA target using RPA is paired with direct detection by *Lachnospiraceae bacterium* Cas12a (*Lb*Cas12a enzyme, referred to herein as Cas12a). A Cas12a workflow permits using fewer reaction components than a Cas13a workflow because the enzyme can directly detect the RPA product, eliminating the need for in vitro transcription (Fig. 1A). We began by designing and testing a set of six RPA primer and Cas12a crRNA sets, targeting the multicopy insertion sequences *IS6110* and *IS1081*, found uniquely within the member species of the *Mtb* complex (MTBC) (for details, see Methods).

To compare the sensitivity of each design for both amplification and detection while minimizing their effect on each other, we carried out RPA for 30 min before spiking Cas12a into the reactions (Fig. 1B and fig. S2A). Primer-crRNA designs cr2 and cr5 outperformed our other assays, detecting 100 copies of the synthetic insertion sequence per microliter of the input synthetic DNA (Fig. 1B). As cis cleavage of the target amplicon in a one-pot reaction interferes with further amplification (*41*, *42*), we aimed to maximize the potential for RPA to overcome the rate of Cas12a cis cleavage. We varied the concentration of Cas12a in one-pot and spiked-enzyme conditions targeting synthetic copies of the insertion sequence, finding that lower concentrations of Cas12a improved the sensitivity of the one-pot assay (Fig. 1C and fig. S2B). Design cr5 appeared to be the most effective combination of primers and a crRNA in these improved conditions for one-pot detection targeting the synthetic insertion sequence (Fig. 1D and fig. S3A). Even with conditions modified to improve assay sensitivity, the one-pot format still performed less sensitively than did the initial spike-in experiments, as seen for designs cr2, cr4, and cr5, possibly due to incomplete amplification (Fig. 1, B and D).

The analytical sensitivity of the Cas12a assay (design cr5) was determined using genomic DNA from *Mtb* H37Rv, the most predominantly used reference strain of *Mtb* for experimental testing, and specificity was evaluated against 10 nontuberculous mycobacteria (NTMs) and other pathogens (*43*). The H37Rv genome contains 17 copies of *IS6110*, and, despite the added complexity of the genomic material as compared to synthetic copies of the insertion sequence, our one-pot Cas12a cr5 assay could detect H37Rv DNA at 10 genomes per microliter (*IS6110* at ~170 copies per microliter) in 100% (three of three, *P*_adj_ < 0.0001) of the technical replicates and could detect down to 1 genome per microliter in two of the three (66%, *P*_adj_ < 0.05) technical replicates (Fig. 1E and fig. S3B). Design cr5 showed no cross-reactivity with NTMs and other pathogens at 1 ng/μl per test as all bars except *Mtb* were found to be not significant (n.s.) compared to nontarget control (NTC; Fig. 1F and fig. S3C).

### Cas13a assay for *Mtb* detection overcomes Cas12a limitations

We reasoned that the decreased sensitivity of the Cas12a-based assays in a one-pot format seen in Fig. 1D compared to that in Fig. 1B was due to Cas12a cleaving either the target DNA in cis or the RPA primers in trans before sufficient amplification could occur, thereby reducing exponential target amplification and trans cleavage of the reporter (Fig. 1A). As Cas13a requires an RNA target and cleaves RNA in trans, we hypothesized that Cas13a would provide higher sensitivity in a single-pot reaction as it would not disrupt amplification in the same manner as Cas12a despite requiring the added complexity of in vitro transcription in the one-pot reaction.

**Fig. 2.**
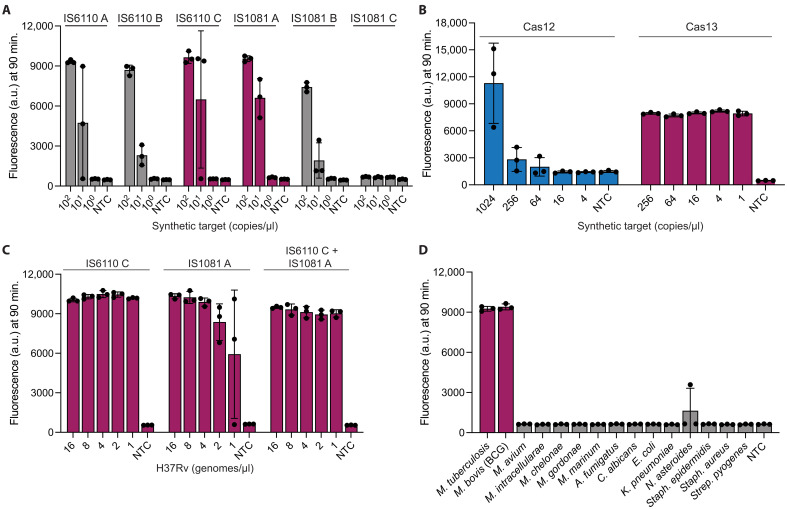
CRISPR-Cas13a–based detection of *Mtb*. (**A**) Cas13a detecting synthetic targets for six different crRNA-primer designs against two different targets, *IS6110* and *IS1081*. (**B**) Comparison of Cas12a to Cas13a detection of *IS6110* synthetic target. Cas13a is a single-crRNA assay for direct comparison. (**C**) Cas13a detecting H37Rv in single-target detection [*IS6110* only; *P*_adj_ < 0.0001 for all bars or IS1081 only; *P*_adj_ (left to right) is <0.001, <0.001, <0.001, <0.01, and <0.05] and dual-target detection (*IS6110* and *IS1081*; *P*_adj_ < 0.0001 for all bars) formats. (**D**) Cross-reactivity panel for Cas13a dual-detection assay against various NTMs and other pathogens at 0.1 ng/μl. All samples are n.s. except for positive controls. In all panels, error bars: SD based on *n* = 3 technical replicates. In (C) and (D), one-way ANOVA with Dunnett’s test was performed and *P*_adj_ values were calculated compared to NTC that contained water. a.u., arbitrary units.

To test this hypothesis, we used Activity-informed Design with All-inclusive Patrolling of Targets (ADAPT) to design six different crRNA sets for *Leptotrichia wadei* (*Lwa*) Cas13a (herein referred to as Cas13a) for two high–copy number insertion sequences, three targeting *IS6110* and three targeting *IS1081* (*44*). We experimentally tested each design with synthetic targets in a one-pot format, identifying IS6110 C and IS1081 A as promising, as both detected at least two of the three technical replicates at 10 copies per microliter and all higher concentrations (Fig. 2A and fig. S4A). Head-to-head comparison using serial dilutions of synthetic targets in Cas12a- and Cas13a-based one-pot *IS6110* assays showed that Cas13a IS6110 C was more sensitive at detection limits of *IS6110* at 1 copy per microliter, whereas Cas12a cr5 began to lose signal at 384 copies per microliter (Fig. 2B and fig. S4B). This demonstrates that Cas13a has higher sensitivity compared to Cas12a when amplification is combined with detection in a single pot. In subsequent experiments, we used Cas13a for *Mtb* detection.

As several strains of *Mtb* have varying copy numbers of these insertion sequences, we further improved the sensitivity of the assay by targeting both *IS6110* and *IS1081* in a dual-detection assay (Fig. 2C) (*45*, *46*). Combining IS1081 A and IS6110 C into one reaction did not appear to hamper either assay’s sensitivity, where *P*_adj_ values for all dual-detection bars were all <0.0001 when compared to NTC (Fig. 2C and fig. S4C). There was no significant cross-reactivity of the combined *IS6110* and *IS1081* dual-detection assay against 13 NTMs and other pathogens at 0.1 ng/μl, as all points but the positive controls were n.s. (Fig. 2D and fig. S4D). One technical replicate for *Nocardia asteroides* appeared to have fluorescence above background, but, upon retesting this pathogen with input of 2.5 ng/μl, there was no signal, suggesting that the assay is highly specific (fig. S4E).

### Adapting dual-detection CRISPR assay (*IS6110* + *IS1081*) for clinical settings

We incorporated an internal control targeting human DNA into our diagnostic system to (i) to avoid reporting false-negative results due to potential interference from sample inhibitors or poor nucleic acid extraction etc., and (ii) as sample adequacy control, where it ensures that the target nucleic acid was from a human derived sample. After evaluating several targets, we included a Cas12a-based internal control that targets the long terminal repeat of the human endogenous retrovirus-K (ERVK), as each human chromosome carries between 40 and 400 copies of the element (fig. S5) (*47*). Due to the target’s abundance in human DNA, we were able to rely on detection without reducing the concentration of Cas12a for our optimized one-pot assay (fig. S5), even for challenging sample types.

**Fig. 3.**
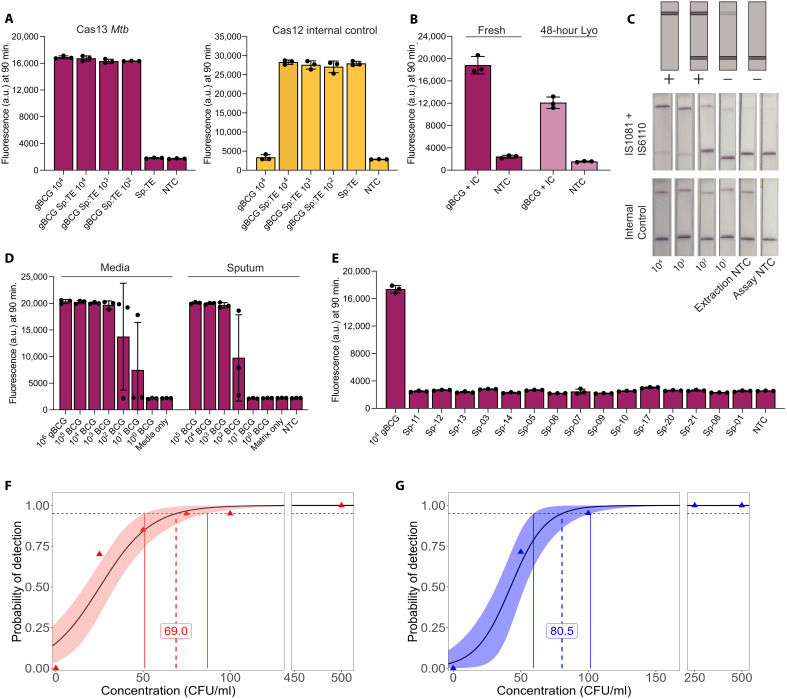
Validation of Cas13a dual-detection assay against clinically relevant samples. (**A**) Cas13a detecting BCG genomic DNA spiked into sputum:TE matrix and Cas12a detecting internal control. Sp is sputum, and gBCG is genomic BCG. Units are genomes per microliter. Cas13a reporter is FAM, and Cas12a reporter is HEX. (**B**) Comparison of fresh Cas13a (left) or lyophilized Cas13a (right) assays detecting synthetic internal control (IC) and BCG genomic DNA. Synthetic IC and genomic BCG were mixed at a concentration of 10^4^ and 10^3^ copies per microliter, respectively. (**C**) Lateral flow readout of Cas13a detecting dual targets *IS6110* + *IS1081* (top) and Cas12a detecting internal control (bottom) in BCG spiked into sputum:TE matrix. Appearance of the top line is a positive readout. Faint lines are interpreted as negative. Disappearance of the bottom line is not necessary. (**D**) Range finding experiment demonstrating the dynamic range of fresh Cas13a dual-detection assay. Cas13a detecting BCG and Cas12a detecting internal control. BCG culture [left; *P*_adj_ (left to right) is <0.001, <0.001, <0.001, <0.01, <0.05, n.s., n.s., and n.s.] and BCG spiked into sputum:TE [right; *P*_adj_ (left to right) is <0.0001, <0.0001, <0.0001, <0.05, n.s., n.s., and n.s.]. gBCG is genomic BCG and units are genomes per microliter. All other units are colony-forming units (CFU) per milliliter. (**E**) Testing fresh Cas13a and Cas12a coupled assay against TB-negative clinical sputum samples. Numbers indicate patient number. All samples are n.s. except Sp-17, where *P* = 0.015. (**F**) LoD for H37Rv. LoD = 69.0 (51.0 to 86.9). (**G**) LoD for BCG. LoD = 80.5 (59.4 to 101.6). In (A), (B), (D), and (E), error bars: SD based on *n* = 3. (C) A single representative sample. (F) *n* = 20 and (G) *n* = 21. In (D) and (E), one-way ANOVA with Dunnett’s test was performed, and *P*_adj_ values were calculated compared to NTC that contained water. a.u., arbitrary units.

To enable SHINE testing of sputum, the standard sample type for pulmonary TB diagnosis, we used a simple and efficient sample processing method developed in parallel by Modi *et al.* (*40*). This method processes sputum samples for detection by our CRISPR assays and other nucleic acid amplification methods (see Methods) while circumventing the need for specialized equipment (*40*). We tested the compatibility of our assay with sputum by spiking *Mycobacterium bovis* Bacille Calmette-Guérin (BCG) genomic DNA from American Type Culture Collection (ATCC) at known concentrations into pooled and diluted sputum samples collected from TB-negative patients at University Hospital in Newark, NJ. As low as 10^2^ copies of the BCG genome per microliter of sputum (the lowest tested concentration in this experiment) were consistently detected by the assay, indicating compatibility with this clinically relevant sample type (Fig. 3A and fig. S6A).

For ease of use, we attempted to combine Cas12a and the dual-detection Cas13a assay in a single SHINE reaction. However, this integration reduced sensitivity and slowed the rate of signal accumulation for both enzymes (fig. S7). We found that excluding any one of the three amplification-detection sets restored this sensitivity, likely the result of competing exponential demands on shared RPA machinery (Fig. 2 and fig. S7A). As sensitivity and sample coverage are critical to the utility of any diagnostic assay, we opted to split the Cas12a internal control and Cas13a dual-detection assay into two chambers, one with the internal control and one dedicated to *Mtb* detection. As these assays are run from the same processed sample and performed in parallel, the separation of components should minimally increase labor. Herein, this parallelized assay format is referred to as SHINE-TB.

As TB cases are most prevalent in low- and middle-income countries, it is critical to develop accessible and field deployable assay format. Similar molecular diagnostics have previously been shown to be amenable to freeze-drying with minimal effects on assay performance (*10*, *16*). We lyophilized single-use tubes of SHINE-TB for 24 hours and stored them at 4°C for 48 hours before testing the resuspended assays on BCG genomic DNA mixed with synthetic internal control, yielding comparable results to their fresh assay counterparts, albeit with a reduction to maximum fluorescence in this fluorometric assay format (Fig. 3B and fig. S6, B and C). We also converted the reporter to be compatible with a lateral flow readout, whereby the quencher at one end of the reporter is replaced by biotin; intact reporters are immobilized at a lower streptavidin band while the antigenic ends of cleaved reporters freely migrate past to reach the upper band (disappearance of the lower biotin line is not required; faint positive test lines are interpreted as negative; Fig. 3C and fig. S8). When using BCG-spiked sputum, the assays consistently detected the internal control and BCG at 10^2^ colony-forming units (CFU)/ml like its fluorometric counterpart. Quantifying band intensity using Fiji showed strong positives through BCG at 10^2^ CFU/ml spiked into sputum:TE matrix and strong positives for all samples for the internal control (fig. S8).

### Characterizing assay performance in contrived clinical samples

To determine the dynamic range of the final fluorometric SHINE-TB assay, we tested pooled sputum samples spiked with BCG cultures at various concentrations. BCG culture was used as positive control. We found that sputum compared to medium did not change the upper limit of detection (LoD) but did affect the lower limit of the Cas13a dual-detection assay, where the samples with BCG at 10^2^ CFU/ml in pooled samples [representing between 0 and 5 copies of *IS6110* per assay, based on digital PCR (dPCR)] appeared to be near the threshold of detection (Fig. 3D, tables S2 and S3, and fig. S9A). But both are still significantly different from the NTC, with *P*_adj_ values of <0.05. Internal control signals remained unchanged irrespective of background BCG concentrations (fig. S9, B and C).

We then tested the specificity of fresh SHINE-TB against individual sputum samples from 14 TB-negative patients to mimic real-world sample variability. Sputum samples were collected from TB unsuspected patients with respiratory symptoms and were confirmed *Mtb* negative using dPCR (Fig. 3E). The *Mtb*-negative samples did not elicit any false-positive assay detections, although two samples, Sp-11 and Sp-09, tested negative for the internal control, suggesting variable sample extraction or assay inhibition (fig. S9D). Additionally, one sample, Sp-17, had a *P*_adj_ value of <0.05 but >0.01, which corresponded to only a 19.4% increase in fluorescence compared to NTC. Of note, Sp-10 was a separate sputum sample collected from the same donor as Sp-09, which did not inhibit the assay, showing that there is variability even in samples collected from the same patient.

The LoD of the Cas13a dual-detection assay was established with various concentrations of BCG (50 to 750 CFU/ml) and H37Rv (25 to 500 CFU/ml) spiked into confirmed *Mtb*-negative pooled sputum; DNA was extracted using the sample processing described in Methods (*40*). LoD fluorescence cutoffs were established as the average fluorescence of the sputum-only controls plus three times their SD. The LoD was defined as the concentration at which this threshold was reached by 95% of the replicates at a single concentration, as inferred by a logistic regression curve. The LoD for BCG was determined to be 80.5 [*n* positive/*N* total, 59.4 to 101.6, 95% confidence interval (CI)] CFU/ml (Fig. 3F and fig. S10, A and E). All concentrations above 100 CFU/ml were detected, and 15 of the 21 samples at 50 CFU/ml were detected. At 50 CFU/ml, there is *IS6110* at ~1 copy per microliter in the input sample, reaction at ~2 copies per 20 μl, according to dPCR (table S4). Note, due to the low copy number and high viscosity of sputum, samples containing below 500 CFU/ml required mixing by pipetting 10+ times. All samples were positive for the internal control (fig. S10B). The LoD for H37Rv was 69.0 (51.0 to 86.9, 95% CI) CFU/ml (Fig. 3G and fig. S10, C, F, and G), and all samples were detected by the internal control assay (fig. S10D).

### SHINE-TB demonstrates high clinical sensitivity and specificity

We performed a clinical validation of SHINE-TB on 14 sputum samples collected from a convenience sample of symptomatic adults undergoing evaluation for pulmonary TB in two public primary healthcare networks in Cali, Colombia. Median age was 44 years old (range, 32 to 59), 28.6% (4 of 14) were female, and 7.1% (1 of 14) were HIV-positive with a mean cough duration of 30 days (range, 20 to 60). Samples were characterized by smear microscopy, Mycobacteria Growth Indicator Tube (MGIT, liquid culture), and GeneXpert MTB/RIF Ultra (“Ultra”) (table S5). Among the 14 samples, 6 were MGIT culture positive (all smear microscopy positive with low-high positive Ultra results), 7 were MGIT culture negative, and 1 was contaminated (indeterminate culture status).

**Fig. 4.**
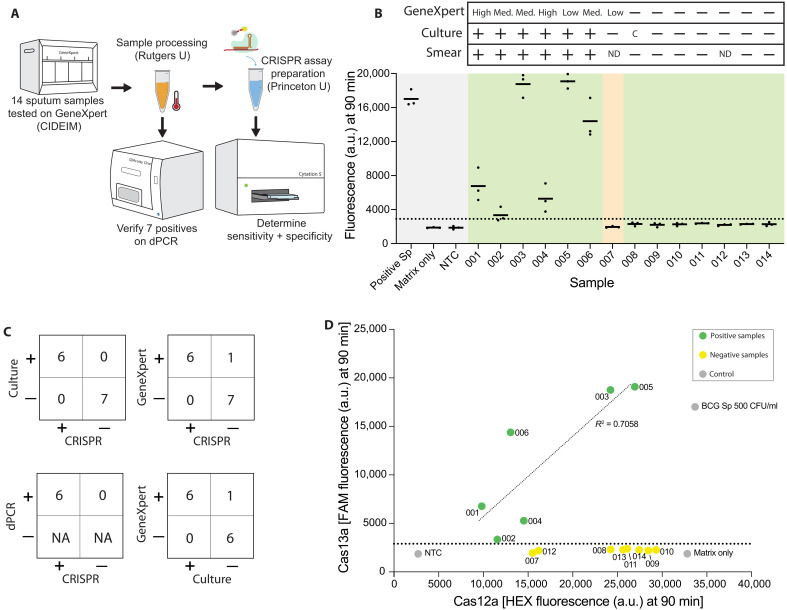
TB detection from sputum using fresh SHINE-TB. (**A**) Schematic showing workflow of patient sample testing. Sputum samples were collected and tested using GeneXpert at CIDEIM in Colombia. Samples were shipped to Rutgers for sample processing. Processed samples were used for CRISPR assays and dPCR. (**B**) TB detection in 14 different sputum samples using Cas13a dual detection. For each sample, a line is shown at the mean of *n* = 3 technical replicates. Shading: gray, controls; green, correct call; yellow, unmatched call for GeneXpert versus CRISPR and culture. LoD is shown as a dotted line at 2900 a.u. Triplicates were averaged for determination of positive and negative. Positive sputum is 500 CFU/ml. Plus signs mean positive detection, and minus signs mean no detection. C means contamination/not determined. ND, not done or data not collected. (**C**) Tables showing Cas13a dual detection compared to culture (top left), GeneXpert (top right), and dPCR (middle right). Table showing GeneXpert versus culture (bottom right). For further details, see table S5. NA, data not collected. (**D**) Average Cas13a (FAM) versus Cas12a internal control (HEX) signal for each sample seen in (B). Linear trend was determined for the positive samples with a coefficient of determination (*R*^2^) value of 0.7058. a.u., arbitrary units.

The samples were processed using our sample processing method described in the methods for use in SHINE-TB and dPCR assays (Fig. 4A). SHINE-TB identified all six culture-positive samples as positive (100% sensitivity) and all seven of the culture-negative samples as negative (100% specificity) (Fig. 4B, fig. S11, and table S6). This compared favorably to Ultra, which showed 100% sensitivity (six of six) and 86% specificity (six of seven; one Ultra low-positive, culture-negative sample) against the single MGIT culture (Fig. 4, B and C, and table S5). The one sample with a contaminated/indeterminate culture was negative by smear, Ultra, and SHINE-TB. For further information on the samples, see table S5.

We also plotted the signal intensity for our *Mtb* assay versus internal control assay (Fig. 4D). For the samples with positive *Mtb* signal, there was a positive correlation between Cas13a signal and Cas12a signal, supporting that internal control performance reflects the performance of the *Mtb* assay and, thus, may help indicate issues with sample inhibition.

## DISCUSSION

We have demonstrated the accuracy and feasibility of SHINE-TB for the detection of *Mtb* directly from clinical samples. We developed and compared two separate Cas12a- and Cas13a-based assays for the detection of MTBC, finding that Cas13a performs better for the purposes of one-pot, manipulation-free diagnostic assays. As high-burden TB countries often face substantial resource limitations, isothermal techniques provide equipment free ways to amplify nucleic acids. Loop-mediated isothermal amplification is most widely used isothermal amplification method but requires incubation at 60° to 65°C for the duration of the reaction and is known to occasionally produce false-positive results because it relies on numerous long primers that each have a nonnegligible probability of off-target hybridization (*22*, *24*, *48*–*51*). RPA can amplify sequences at 37°C using only a pair of primers per amplicon but can be subject to nonspecific amplification when longer primers are used for more efficient reactions, while the integration of CRISPR with RPA may help mitigate these issues with an added layer of specificity checking (*23*, *25*, *39*, *52*–*54*).

Our assays were designed to create a one-pot procedure that does not require a mechanical separation between isothermal amplification and detection. Despite integrating suboptimal PAM sequences to mitigate the competition between Cas12a and amplification (*42*), we found that Cas13a had greater sensitivity than Cas12a. Cas13 has been favored in other studies as well, possibly due to its higher turnover for trans-cleavage (*14*, *16*, *33*, *55*, *56*).

We then expanded this to a Cas13a dual-detection assay for other strains of *Mtb* with varying *IS6110* and *IS1081* copy numbers (*45*, *46*, *57*). Although we favored Cas13a for a one-pot test that required sensitivity and robustness, Cas12a exhibited appropriate sensitivity for an internal control targeting a highly abundant genetic element in properly extracted human samples, as standard samples had substantial signal saturation.

We have demonstrated the feasibility of lyophilization and lateral flow readout to provide a means for deployability to lower resource areas. If the two were combined and considering the robust sample preparation, then this would allow for minimal sample handling with minimal equipment. For ease of use in the field, it may be important to include a color reference visual scorecard for user interpretation of positive or negative lateral flow results in order to mitigate user bias (*58*). Entirely equipment-free SHINE is possible as 37°C is the optimal temperature for both RPA and Cas13a (*16*). Previous CRISPR-based TB studies have successfully incorporated lateral flow (*29*, *31*–*33*, *36*), but not in a one-pot format. SHINE-TB is the only TB test where amplification and detection are combined that is compatible with both lateral flow and lyophilization.

SHINE-TB uses much less sputum sample volume (100 μl per extraction and ~2 μl per reaction) compared to GeneXpert MTB/RIF cartridges (1 ml) (*59*). Despite contributing just 10% of the overall reaction volume, some individual sputum types showed clear assay inhibition for both Cas13a and Cas12a using the crude sample prep, possibly due to inhibition of RPA (*60*). Additional experiments are required to fully understand the mechanisms of inhibition; however, if the internal control also fails, then the test samples will be called “indeterminates,” requiring repeat testing. For one negative patient sputum sample (Sp-17), we found it to be statistically significant compared to the NTC when the *P*_adj_ cutoff was 0.05 but n.s. if the *P*_adj_ cutoff was 0.01 (Fig. 3E). As sputum is inherently variable as a matrix and accuracy for clinical diagnosis is vital, we consider that a *P*_adj_ value of 0.01 would be reasonable to prevent false positives and do not find this point to be of concern.

SHINE-TB has comparable performance to other gold-standard methods for detecting TB DNA. Our optimized Cas13a SHINE-TB assay had an LoD of ~80 CFU/ml for BCG spiked in sputum, which was comparable or better than the published Ultra BCG LoD of 143.4 CFU/ml (*61*). For H37Rv spiked in sputum, we determined an LoD of ~70 CFU/ml, which was slightly higher than the published Ultra H37Rv LoD of 15.6 CFU/ml (*61*). Considering the expected higher copy numbers of both *IS6110* and *IS1081* in *Mtb* H37Rv compared to *Mycobacterium bovis* BCG, we expected the H37Rv LoD to be much better (fig. S10, E to G). Because dPCR testing of BCG at 50 CFU/ml detected IS6110 at ~1 copy per microliter, the target genomes may follow a Poisson distribution, meaning some reactions may randomly contain no genomes at low concentrations (table S4). Thus, at these concentrations, increased copy number may not increase detection. Additionally, other isothermal amplification–based *Mtb* assays showed similar sensitivity, with LoDs of 50 to 100 CFU/ml (*27*, *30*, *62*). SHINE-TB detected *Mtb* in a small set of smear-positive clinical sputum samples with 100% specificity and sensitivity compared to culture. These results meet the World Health Organization’s (WHO) target product profile (TPP) benchmarks of ≥85% sensitivity for near POC and ≥90% sensitivity for low complexity tests for TB (*63*). While this preliminary clinical evaluation is encouraging, larger field-based evaluations involving diverse participants and smear-negative, culture-positive samples are needed for more comprehensive and precise performance measures.

Additional steps must be taken to address broader needs for the field. While our current extraction method requires minimal instrumentation, it still relies on low-speed benchtop centrifuge. In settings without reliable power supply, alternative methods, such as hand-powered paper centrifugation, could be explored as an alternative to a table-top centrifuge (*64*). As drug-resistant strains of *Mtb* become increasingly prevalent, many diagnostics include drug susceptibility testing (*61*, *65*). Preferably, a diagnostic would be able to identify resistance for first-line drugs, such as rifampicin. Increased multiplexing is necessary to achieve this goal, as current amplification restrictions prevent us from amplifying more than two targets. In the future, it would be useful to increase multiplexing capacity by changing amplification strategies or by spatially isolating parallel reactions (*15*, *56*, *66*). Additional improvements would expand the compatibility of the assay to multiple sample types, as sputum can be difficult for some patients to produce (*67*). Ideally, our platform would be compatible with sample types such as saliva and oral swabs (*68*, *69*). Further testing will be needed to determine which sample types are amenable to extraction and diagnosis, while better biochemical characterizations should help identify compounds that are inhibitory to the overall diagnostic process. Our simple one-pot assay provides robust *Mtb* detection, taking us one step closer to accessible and reliable TB diagnostics.

## METHODS

### Synthetic targets and genomic DNA

Gene fragments for *IS6110* and *IS1081* were ordered from Integrated DNA Technologies (IDT) (sequences listed in table S7) and resuspended in nuclease-free (nf) water. BCG genomic DNA was ordered from ATCC (catalog no. 35734D-2) and quantified with dPCR. Quantitative *Mtb* genomic DNA was ordered from ATCC (catalog no. 25177DQ).

### Sequence design

Cas12a target sequences were chosen by first compiling canonical sequences and related diversity information for chosen insertion sequences or possible internal control genes using BLAST (*70*) and Clustal Omega (*71*) on publicly available datasets. We then annotated these sequences for all canonical and permitted noncanonical Cas12a PAM sequences and ranked candidates manually for conservation throughout the amplified region and the energetics of crRNA ensemble conformations (*72*). Cas13a designs were generated using ADAPT on the same consensus sequences with 40-nucleotide primers and, otherwise, default parameters, before being manually ranked for conservation and free energy, as above (*44*). The Cas13a crRNA IS6110 C had a slightly lower ADAPT rating than the other chosen candidates but was made to overlap with Cas12a cr5 to compare the two assays’ efficiencies more directly. A T7 RNA polymerase promoter was added to the 5′ end of all forward primers for Cas13a assays. All crRNAs, coupled primers, and synthetic targets used in this publication are listed in table S8. 

### Two-pot and one-pot Cas12a assays

Two-pot Cas12a assays were performed by mixing an amplification master mix (below) with target, allowing amplification to occur for 30 min before adding Cas12-crRNA RNPs (Fig. 1, B and C). Master mixes (concentrations given for final reaction) were generated by combining SHINE buffer [20 mM Hepes (pH 8.0); 60 mM KCl; 3.5% polyethylene glycol (PEG-8000); and nf water], ribonuclease inhibitor (1 U/μl; New England Biolabs, M0314L), 70 nM of each RPA primer (IDT), 0.25 μM fluorescein (FAM) 5C quenched reporter (IDT), and 14 mM MgOAc in nf water. TwistAmp Basic RPA pellets (TwistDx Limited, TABAS03KIT) were used for amplification, whereby one pellet was resuspended for every 107.5 μl of final reaction volume. The target (10% of final reaction volume) and amplification master mix were combined, lightly vortexed, and incubated at 37°C for 30 min in an Agilent BioTek Cytation 5 microplate reader, taking fluorescence readings every 5 min (excitation, 485 nm; emission, 525 nm). crRNAs (IDT) and *Lb*Cas12a Ultra (IDT, 10007922) in nf water at 10× their final equimolar concentration of 4 or 20 nM were combined on ice for 15 to 20 min and added to each reaction 30 min into the reaction. Reactions (15 μl) were performed in technical triplicates (with the exception of two early optimization experiments performed in duplicate to increase condition throughput) in 384-well clear-bottom microplates (Greiner, 788096). Reactions were then returned to the plate reader at 37°C for up to 3 hours of additional fluorescence readings.

One-pot Cas12a assays were performed by mixing complete Cas12a master mix (90% by volume) with target (10% by volume) (Figs. 1, C to F, and 2D). Master mixes were prepared as in the two-pot protocol, except that crRNAs and Cas12a were added in the master mix before adding target, for a final concentration of 4 nM (Figs. 1 and 2), 30 nM (Fig. 3), or as otherwise indicated. Final reactions were performed as stated above.

### One-pot Cas13a assays

One-pot Cas13a assays were performed as for the one-pot Cas12a reactions, except that, to form the Cas13a master mix, the same SHINE buffer and 14 mM MgOAc were mixed with 45 nM *Lwa*Cas13a [GenScript, stored in 100 mM tris HCl (pH 7.5) and 1 mM dithiothreitol], 0.3 mM of each ribonucleotide triphosphate (rNTP) (New England Biolabs, N0466L), T7 RNAP (1 U/μl; Biosearch Technologies Inc. (Lucigen, NC2089983), 62.5 nM FAM 6U quenched reporter (IDT), 45 nM of a single crRNA (Fig. 2, A to C) or 22.5 nM of each crRNA in a combination (Figs. 2, C and D, 3, and 4), and 70 nM of the appropriate RPA primers in nf water. Reactions were loaded in technical triplicates into Greiner 384-well clear-bottom microplates for 15 μl of reactions (Fig. 2) or Corning 384-well microplates for 20 μl of reactions (Figs. 3 and 4). The reaction was then incubated at 37°C for up to 3 hours in an Agilent BioTek Cytation 5 microplate reader, as above. One-pot Cas13a assays were performed by mixing complete Cas13a master mix (90% by volume) with target (10% by volume). When the target is sputum, pipette mix 10+ times and pulse vortex before spinning down.

Combined Cas12a/Cas13a assays were performed by combining all the nonredundant reagents from the two independent one-pot assays and replacing the DNA reporter with 0.25 μM HEX 5C quenched reporter (IDT), to be measured in two fluorescence channels: excitation, 485 nm; emission, 525 nm; and excitation, 530 nm; emission, 570 nm.

### Lateral flow readouts

Lateral flow methods were based on a previously described protocol (*16*), with some minor modifications. The assay was adapted to lateral flow with the following conditions: the quenched Cas13a reporter was replaced with 750 nM FAM 14 U Bio (IDT) and the Cas12a reporter was replaced with FAM 5C Bio (IDT). Reactions were incubated in PCR strip tubes at 37°C for 90 min before being diluted 1:4 in HybriDetect Assay buffer (Milenia Biotec, MGHD 1). The reaction was equilibrated for 5 min before adding HybriDetect lateral flow strips for 1 min. Images were taken with a smartphone 1 min after dipstick removal. Test line intensity was analyzed using Fiji version 2.14.0/1.54p. Briefly, rectangular boxes were drawn around each test line strip of the same size. Intensity curves were created for the entire area of the rectangle, which included the adjacent pixels above, below, and to either side of the test line. A line was drawn under each peak to isolate intensity associated with the test line. The area under the peak was then quantified using the Wand tool. Values were subsequently plotted in Prism.

### Lyophilization

Lyophilization methods were based on a previously described protocol (*16*), with some minor modifications. The dual assay was optimized to ensure integrity after lyophilization. The reaction was prepared as described above, except that 20 mM Hepes (pH 8.0) was added in place of the complete SHINE buffer. Sucrose at 5% (w/v) and mannitol at 150 mM were added as cryoprotectants. Assays were aliquoted (12 reactions per aliquot), flash frozen, and lyophilized at −30°C for 24 hours using a Freezone Triad Benchtop Freeze Dryer from Labconco. Aliquots were then vacuum sealed alongside a desiccant and stored in 4°C until further use. Assays were resuspended in 3.5% PEG-8000, 60 mM KCl, and 14 mM MgOAc in nf water, aliquoted, and mixed with target. Reactions were loaded in technical triplicates in Corning 384-well microplates for 20 μl of reactions (Fig. 3). Reactions were then incubated at 37°C for up to 3 hours. Fluorescent measurements were taken every 5 min in an Agilent BioTek Cytation 5 microplate reader.

### Collection of clinical samples

TB-negative samples for spiking were collected from patients of University Hospital, Newark, NJ, USA, after written informed consent (Rutgers eIRB no. Pro2020001138). Eligible participants were adults >18 years old diagnosed with a non-TB respiratory condition (e.g., cardiogenic pulmonary edema, asthma exacerbation, and other bacterial pneumonia) and were willing and able to provide an expectorated sputum sample.

Following written informed consent, symptomatic individuals undergoing evaluation for TB at two primary health centers in Cali, Colombia, provided expectorated sputum for examination. The study protocol was reviewed and approved by the Institutional Review Boards for ethical conduct in research involving human subjects at the Centro Internacional de Entrenamiento e Investigaciones Médicas (CIDEIM, no. 1325). The protocol adhered to both national regulations (Resolution 008430, Ministry of Health, Republic of Colombia, 1993) and international ethical guidelines, including the Declaration of Helsinki and its amendments (World Medical Association, Fortaleza, Brazil, October 2013).

For blinded sample testing, a convenience sample of symptomatic adults aged ≥18 years and undergoing evaluation for TB at two primary health centers in Cali, Colombia, provided two expectorated or induced sputum samples on the same day. One was sent for smear microscopy and mycobacterial culture on liquid medium (MGIT) on-site in the clinical laboratories, while the other was sent for molecular testing using the GeneXpert MTB/RIF Ultra assay at the CIDEIM research laboratory in accordance with the manufacturer’s instructions. Participants were selected the following way: research staff (a nursing assistant or a physician) approached consecutive individuals referred for TB microbiological testing according to the laboratory electronic records from district hospitals located in the central and eastern parts of the city [Empresa Social del Estado (ESE) Centro and ESE Oriente]. In addition, the research staff screened a convenience sample of participants in the emergency, respiratory therapy, and outpatient departments for TB symptoms and encouraged clinicians to refer them for TB diagnostic testing.

### Sample processing

Sample processing was performed as previously described and was summarized in the following (*40*).

*Chelex extraction for sputum samples: For individual patient screening, inhibitory patient screening, and dynamic range extractions*. Spiked sputum (100 μl) was aliquoted into a screw-cap Lysing Matrix B tube (MP Biomedicals, MP116911100); subsequently, 200 μl of Chelex100 resin (InstaGene Matrix, Bio-Rad, 7326030) was added. Samples were vortexed three times for 30 s at 3400 rpm each time, subsequently incubated at 95°C for 30 min, and then centrifuged at 9400*g* [relative centrifugal force (RCF)] for 2 min. Supernatant (~60 to 80 μl) was collected to serve as inputs for the diagnostic assays. For Fig. 3A, genomic BCG was spiked into extraction sputum at various concentrations. Culture conditions for BCG and H37Rv are described in (*40*).

*LoD extractions*. Spiked sputum (400 μl) was aliquoted into a screw-cap Lysing Matrix B tube; subsequently, 800 μl of Chelex100 resin (InstaGene Matrix) was added. Samples were vortexed three times for 30 s at 3400 rpm each time, subsequently incubated at 95C for 30 min, and then centrifuged at 9400*g* RCF for 2 min. Supernatant (~800 μl) was collected.

### Clinical sample testing

Clinical samples were prepared using methods described above. Primers, crRNAs, and master mix are the same as above.

dPCR was performed in the QiAcuity dPCR system using QiAcuity 4× Probe PCR kit and 26k eight-well nanoplate (QIAGEN, 250102 and 250031). Previously published sequences for primers and probes were used (IS6110-I-F, IS6110-I-R, and IS6110-TM) (*61*). For the final reaction volume of 12 μl, 3 μl of 4× Probe PCR Master mix, 0.8 μM concentration of forward and reverse primers, 0.4 μM concentration of probe, and 2 μl of DNA sample were added. Molecular grade, nf water was used to complete the volume. Thermal cycling conditions are as follows: PCR initial heat activation at 95°C for 2 min, followed by 40 cycles of denaturation at 95°C for 15 s, and combined annealing/extension at 60°C for 30 s. DNA samples were tested both directly and at 10-fold dilution in replicates of three. *M. bovis* BCG DNA at 100 genome copies per reaction and nf water were used as positive and negative control, respectively.

### Data analysis

Plots and graphs were generated using Prism software. Statistical analysis was performed using Prism software. One-way analysis of variance (ANOVA) was used, correcting for multiple comparisons using the Dunnett’s test, as multiple samples are compared to the same NTC. Averages of replicates for each sample were compared to the corresponding average of the NTC. The cutoff used was *P*_adj_ < 0.05 unless otherwise specified.

Data were plotted in R using the ggplot2 package. Simple logistic regression was used to model detection probabilities for (i) H37RV and (ii) BCG bacteria as a function of concentration (Fig. 3F). The 95% LoD was estimated as the concentration in which the probability of detection of a positive sample is 0.95. Wald confidence intervals (95%) were generated from the SE of the estimated concentrations.

Assay cutoffs for positive or negative results for clinical samples were derived using a 70:30 train-test split strategy of spiked sputum data with known bacterial concentrations ranging from 0 (*Mtb*-negative samples) to 10^4^ CFU/ml (*Mtb*-positive samples). Receiver operating characteristic (ROC) curves were constructed from a dataset of 115 experiments with known bacterial concentrations. Four approaches were explored for setting the cutoff: (i) the mean of the negative samples plus three times the SD of the negative samples, (ii) the point on the ROC curve corresponding to the maximum Youden index, (iii) the point on the ROC curve corresponding to the WHO 2024 TPP sensitivity standard for a sputum-based near-point-of-care test, and (iv) the point on the ROC curve corresponding to the WHO 2024 specificity standard for all diagnostic tests (table S6). Both the Youden index and the specificity standard approaches gave a cutoff of 2895.42 fluorescent units, which was selected and rounded to 2900 for the final cutoff. This cutoff was then applied to evaluating SHINE performance among the 14 clinical samples. The exact method for binomial confidence intervals was used to compute 95% confidence intervals for sensitivity and specificity estimates.

## Supplementary Materials

### This PDF file includes:

Figs. S1 to S11 Tables S1 to S8 References
