# Supplementary material for: A streamlined CRISPR-based test for tuberculosis detection directly from sputum
Source: Sci Adv. 2025 Aug 6;11(32):eadx2067. doi: 10.1126/sciadv.adx2067 (PMC12327463; doi:10.1126/sciadv.adx2067)
Supplement: Supplementary file 1 — Figs. S1 to S11 Tables S1 to S8 References [file sciadv.adx2067_sm.pdf]

Science Advances
AAAS

Supplementary Materials for

A streamlined CRISPR-based test for tuberculosis detection directly from sputum

Alexandra G. Bell *et al.*

Corresponding author: Cameron Myhrvold, cmyhrvol@princeton.edu

Sci. Adv. **11**, eadx2067 (2025)
DOI: 10.1126/sciadv.adx2067

This PDF file includes:

Figs. S1 to S11
Tables S1 to S8
References

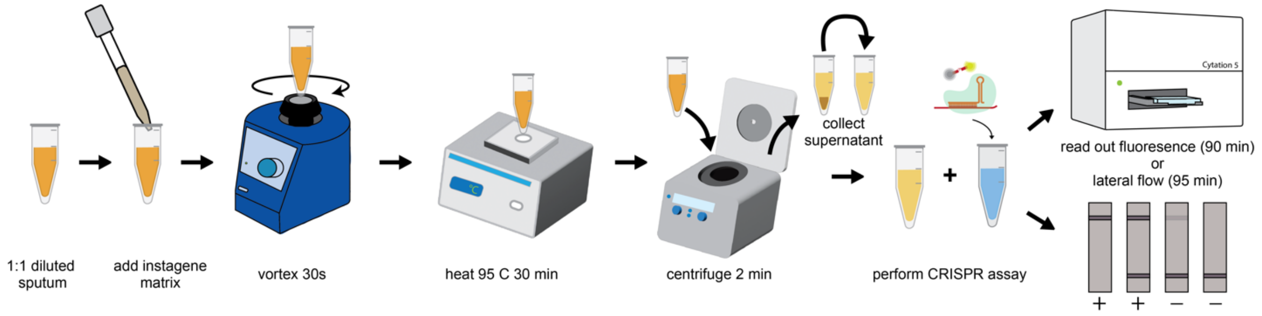

Figure S1. Schematic showing required equipment for extraction through diagnosis in the current implementation of SHINE-TB. The time from collection to readout includes 35 minutes for sample processing, 90 minutes for one-pot amplification/detection, plus an extra 5 minutes for the lateral flow readout, for a total of ~125 minutes. Schematic was redrawn based on Fig. 2 of (40).

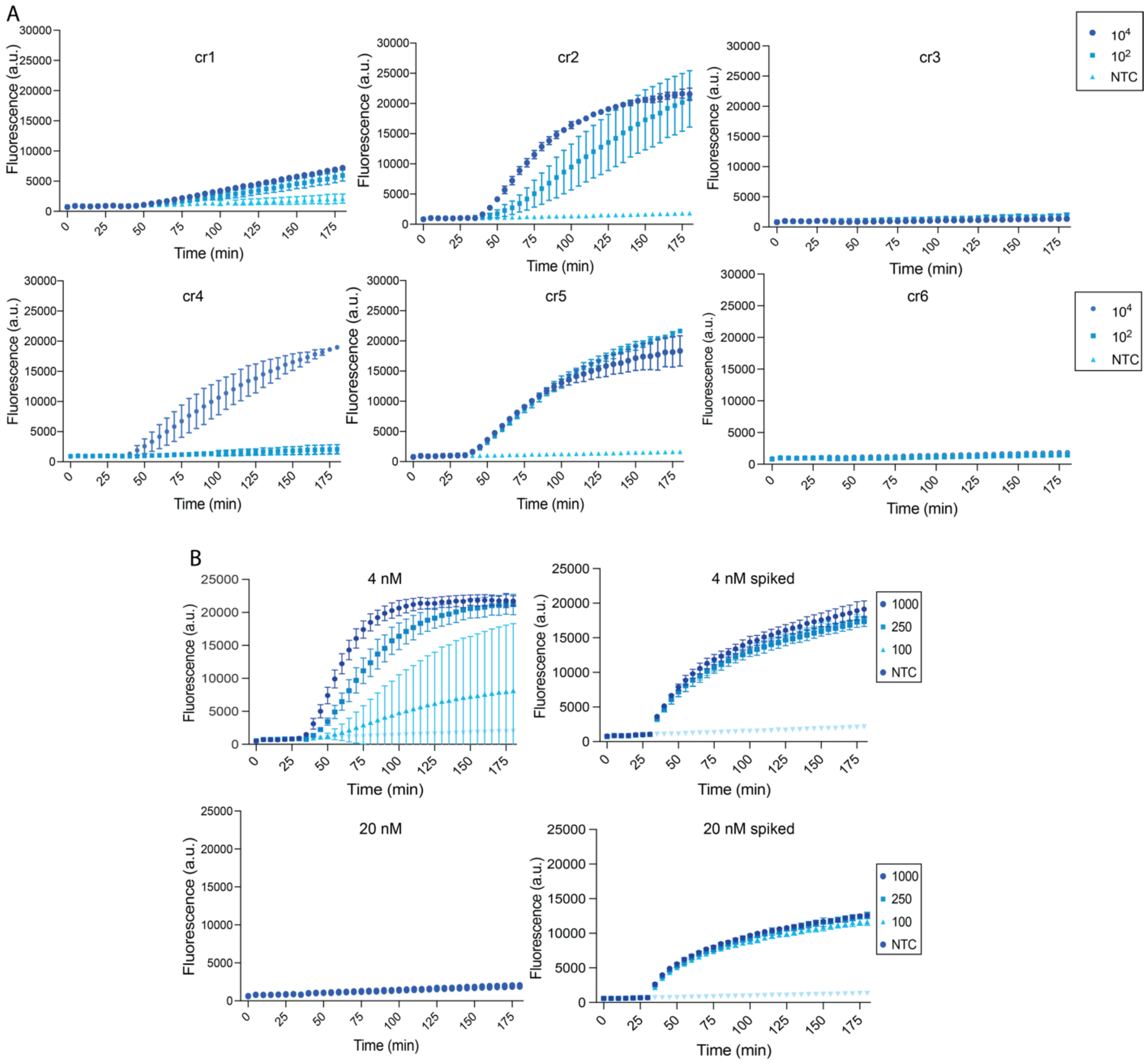

Figure S2. Fluorescence kinetic plots for panels seen in Fig. 1B and 1C. **A.** Fluorescence kinetics from Fig. 1B. The target was synthetic DNA (fragments of IS6110). cr1 (top left), cr2 (top middle), cr3 (top right), cr4 (bottom left), cr5 (bottom middle), cr6 (bottom right). **B.** Fluorescence kinetics from Fig. 1C. The target was synthetic DNA and the Cas12 guide & RPA primer pairing used was cr5. 4 nM (top left), 4 nM spiked (top right), 20 nM (bottom left), and 20 nM spiked (bottom right). In **A**, error bars: SD based on n=2 technical replicates. In **B**, error bars: SD based on n=3 technical replicates. All target concentrations are reported in copies/µL.

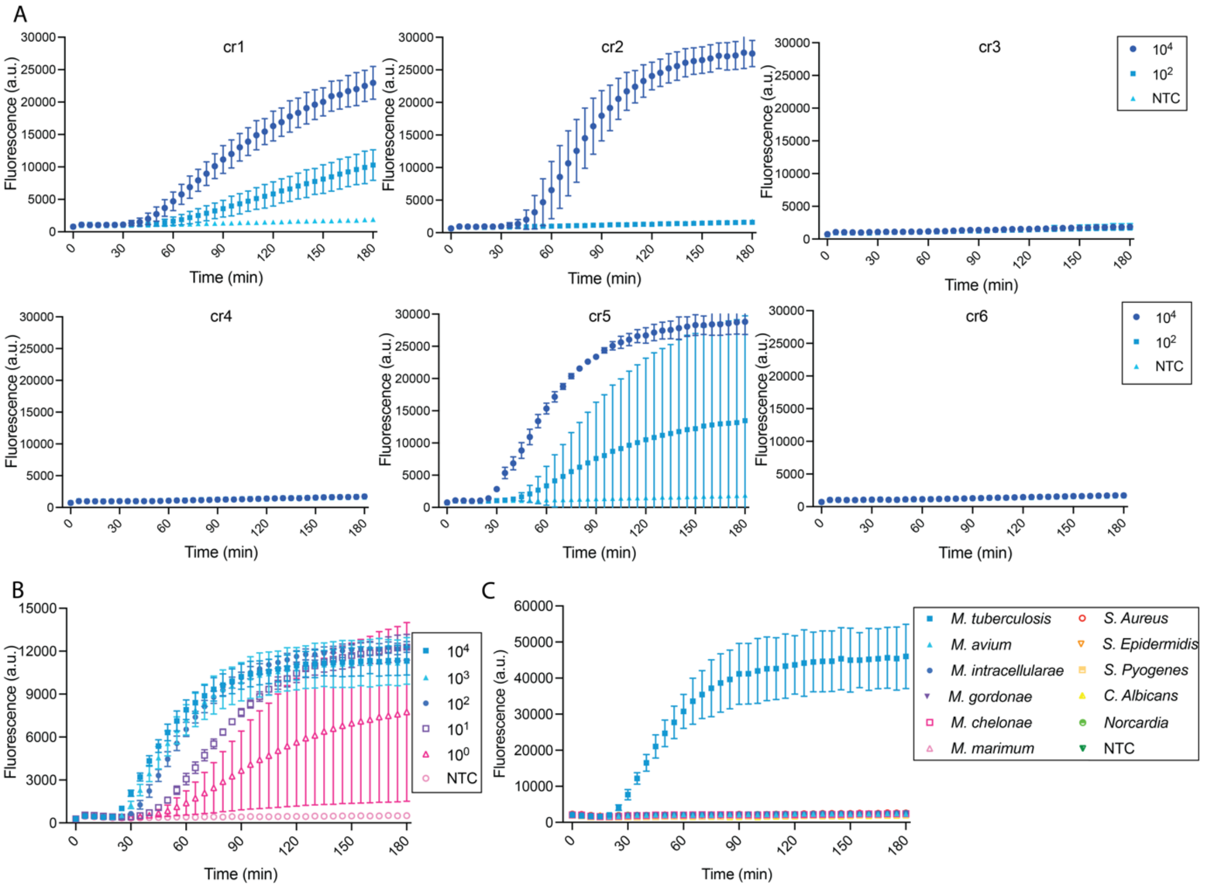

Figure S3. Fluorescence kinetic plots for panels seen in Fig. 1D, 1E and 1F. A. Fluorescence kinetics from Fig. 1D. The target was reported in copies/µL of synthetic DNA. cr1 (top left), cr2 (top middle), cr3 (top right), cr4 (bottom left), cr5 (bottom middle), cr6 (bottom right). **B.** Fluorescence kinetics from Fig. 1E. The target was reported in copies/µL of the H37Rv genome. **C.** Fluorescence kinetics from Fig. 1F, wherein 1 ng/µL of extracted DNA from each microorganism was used as input. In **A**, error bars: SD based on n=2 technical replicates. In **B** and **C**, error bars: SD based on n=3 technical replicates.

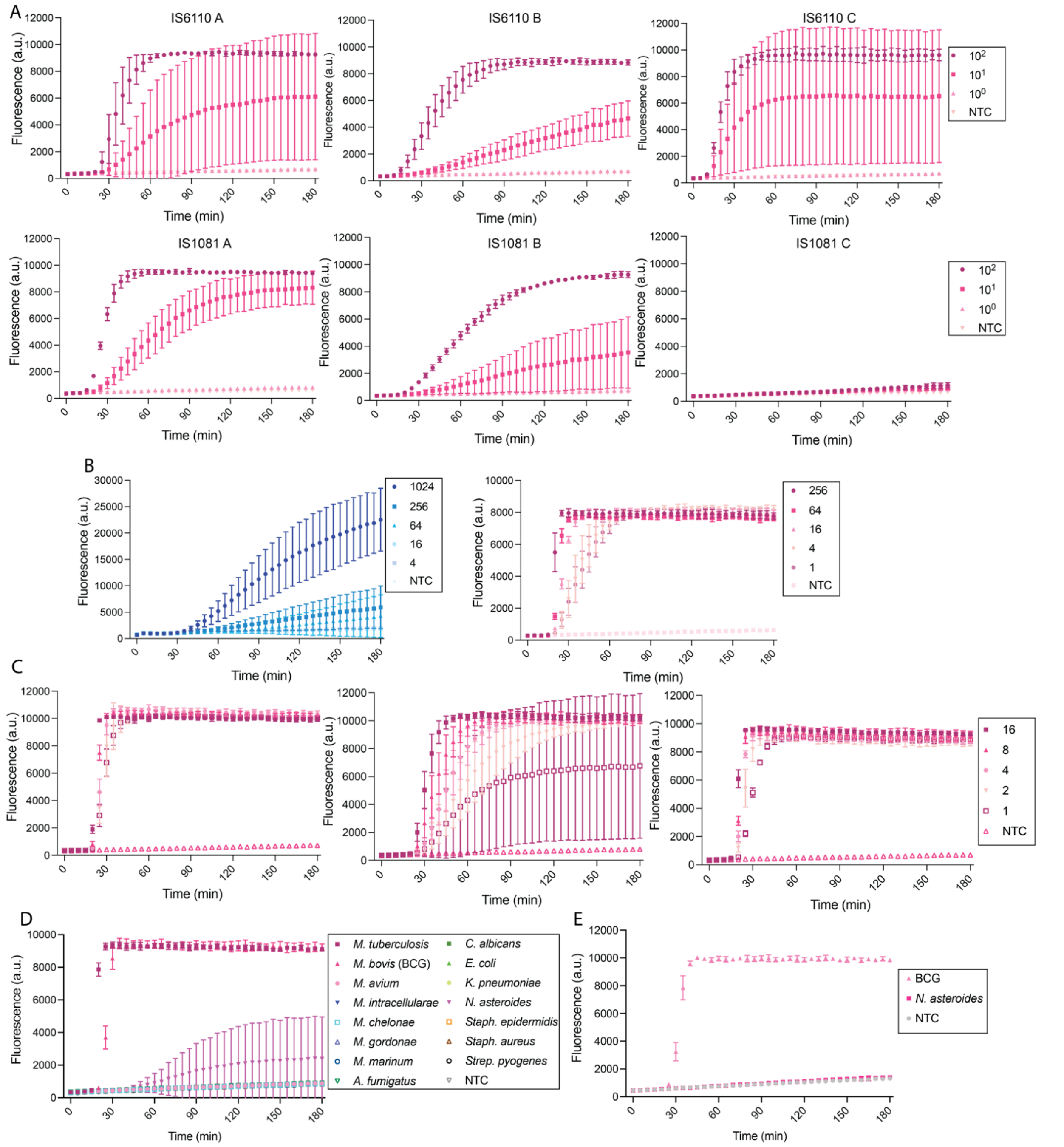

Figure S4. Fluorescence kinetic plots related to Fig. 2. A. Fluorescence kinetics from Fig. 2A. The target was reported in copies/µL of synthetic DNA, paired with the following assays: IS6110 A (top left), IS6110 B (top middle), IS6110 C (top right), IS1081 A (bottom left), IS1081 B (bottom middle), IS1081 C (bottom right). **B.** Fluorescence kinetics from Fig. 2B. The target was synthetic DNA – in this case, a single fragment of IS6110 shared as a target by both assays: Cas12a cr5 (left) and Cas13a IS6110 C (right). **C.** Fluorescence kinetics from Fig. 2C. Serial dilutions of the H37Rv genome were detected using IS6110 C (left), IS1081 A (middle), and their combination (right). **D.** Fluorescence kinetics from Fig. 2D. **E.** Dual-detection Cas13a fluorescence kinetics for *N. asteroides* at 2.5 ng/µL. In all panels, error bars: SD based on n=3 technical replicates.

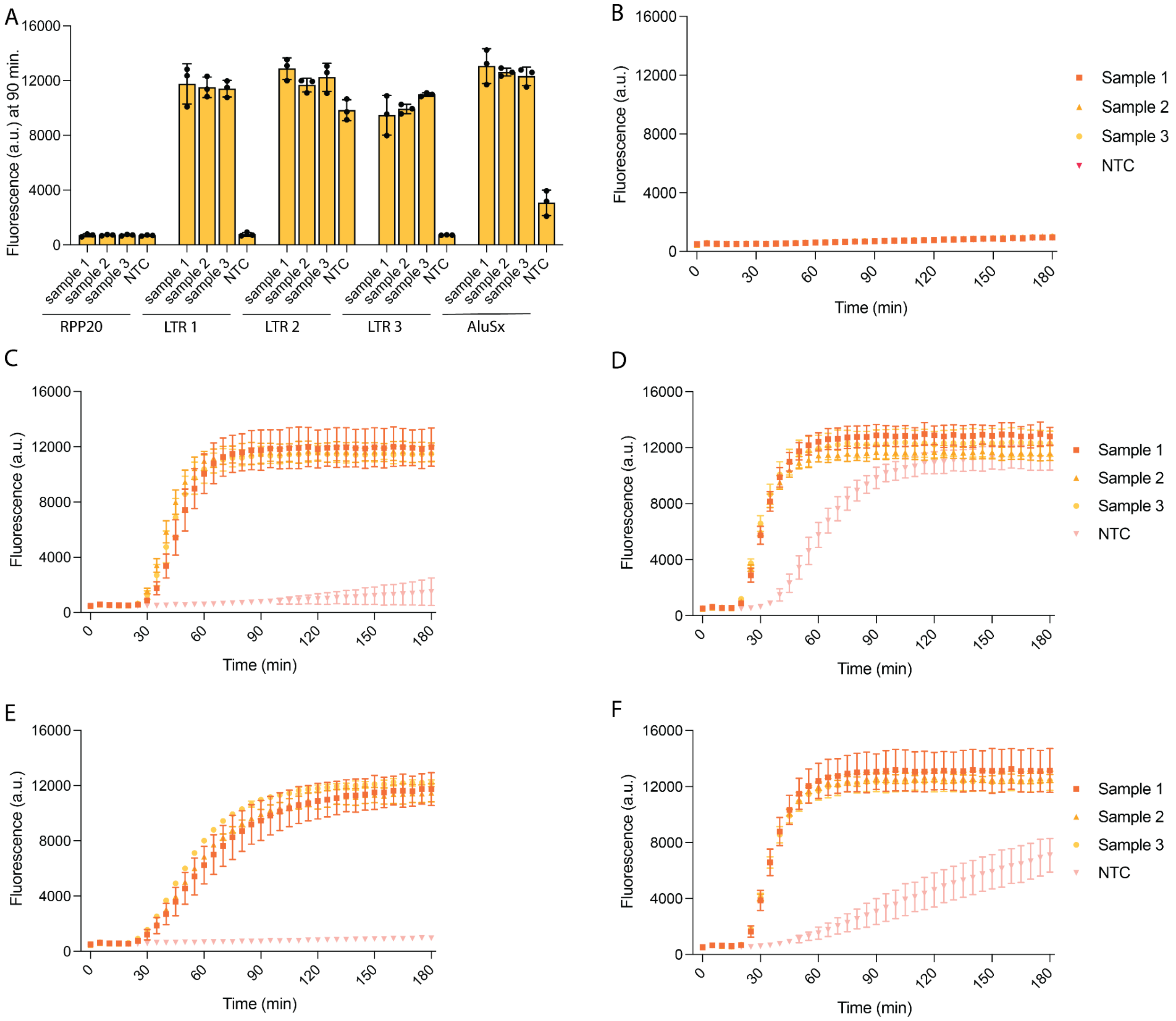

Figure S5. Testing putative internal control targets. A. Guide designs targeting various human endogenous genes. RPP20 (ribonuclease P protein subunit p20), LTR 1-3 (various guides targeting the long terminal repeat of the endogenous retrovirus ERVK), and AluSx (alu transposable element). **B.** Fluorescence kinetics for RPP20 (cross-section at 90 minutes shown in A). **C.** Fluorescence kinetics for LTR (cross-section at 90 minutes shown in a). **D.** Fluorescence kinetics for LTR 2 (cross-section shown in A). **E.** Fluorescence kinetics for LTR 3 (cross-section at 90 minutes shown in A). **F.** Fluorescence kinetics for AluSx (cross-section at 90 minutes shown in A). In all panels, error bars: SD based on n=3 technical replicates. All samples used are pooled human saliva diluted 1:10 in nuclease-free water. Cas12a was included at a final concentration of 20 nM.

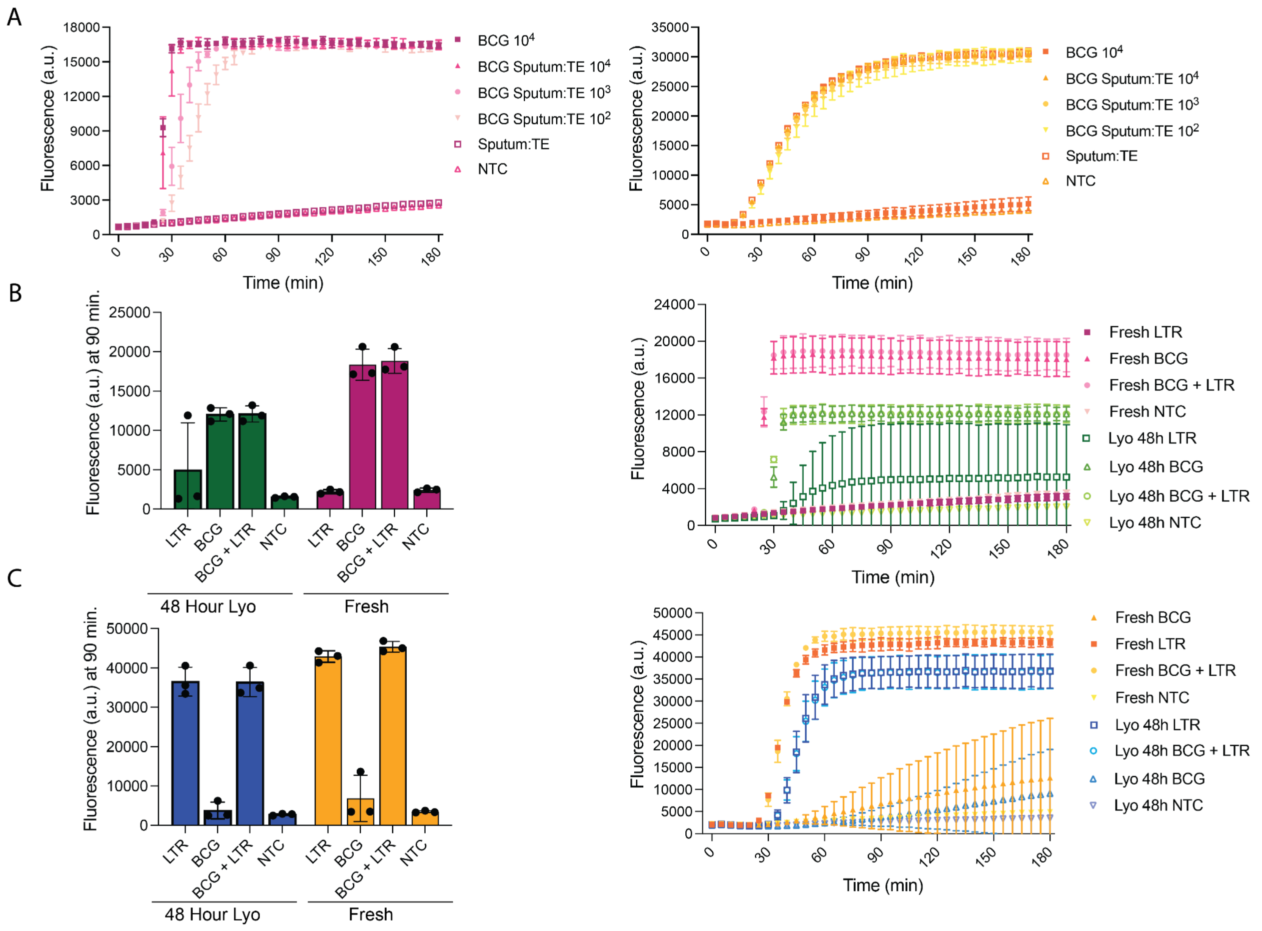

Figure S6. Fluorescence kinetic plots for panels seen in Fig. 3A and 3B. A. Fluorescence kinetics from Fig. 3a. Cas13a *Mtb* dual-detection (left) and Cas12a internal control (right). **B.** Additional hold-one-out controls for Fig. 3B, as reported by the duplexed Cas13a fluorophore (left), alongside relevant fluorescence kinetics (right). A synthetic fragment of the LTR was used at 10^4 copies/µL, while BCG was input to the assay at 10^3 copies/µL. **C.** Additional hold-one-out controls for Fig. 3B, as reported by the Cas12a internal control fluorophore (left). Fluorescence kinetics for internal control data seen in 3B (right). LTR was input at 10^4 copies/µL and BCG was input at 10^3 genomic copies/µL. In all panels, error bars: SD based on n=3 technical replicates.

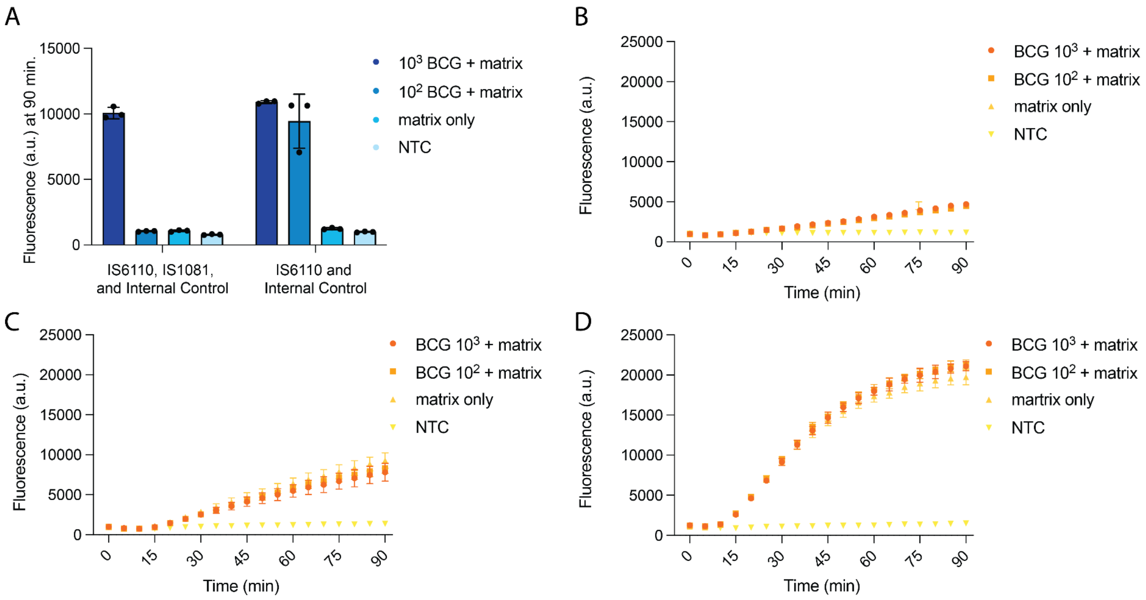

Figure S7. **Trialing assay configurations for single-, dual-, and triplex assays. A.** Comparing the performance of IS6110 C, IS1081 A, and internal control single-pot assays to the combined IS6110 C and IC single-pot assay. Fluorescence at 90 min, as reported by the Cas13a target fluorophore (FAM). **B-D.** Internal control fluorescence kinetics reported by Cas12a target fluorophore (HEX) of: (**B**) the combined IS6110 C, IS1081A, and IC single-pot assay, (**C**) the IS6110 C & IC single-pot assay, and (**D**) the IC single-pot assay. In all panels, error bars: SD based on n=3 technical replicates. In some cases, error bars are smaller than the data points. Matrix was salivary sputum diluted 1:1 in nuclease-free water.

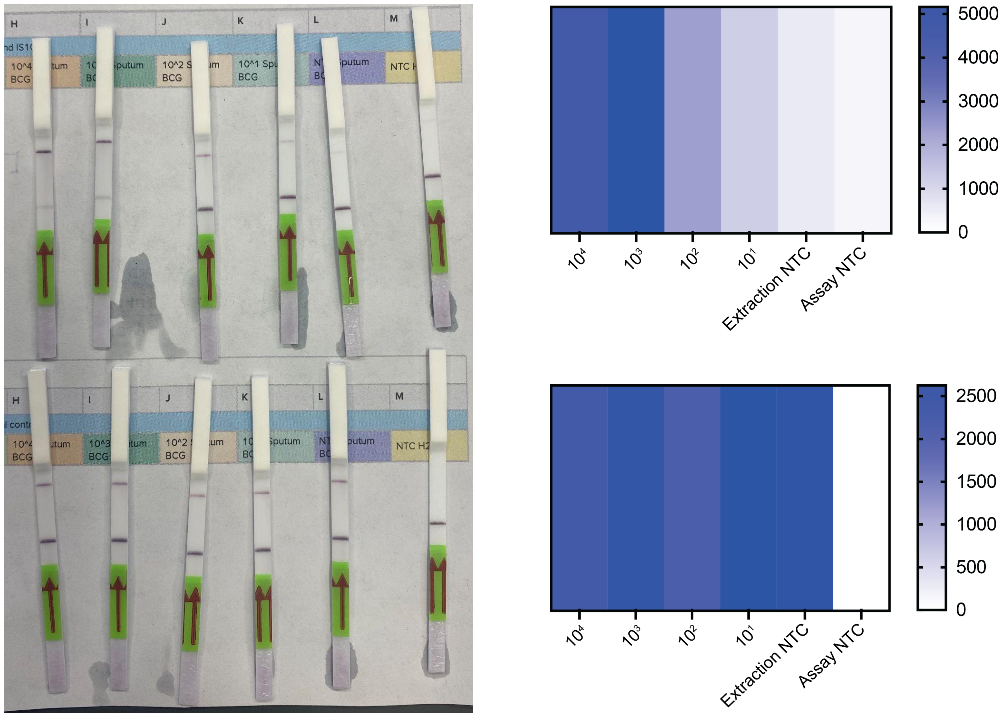

Figure S8. Raw images and relative quantification of lateral flow strips seen in figure 3C. BCG culture in pooled sputum:TE (left). Quantified band intensity for LF test line in raw images (right). Quantification was done using Fiji. Briefly, rectangular boxes were drawn around each test line strip of the same size. Intensity curves were created for the entire area of the rectangle, which included the adjacent pixels above, below, and to either side of the test line. A line was drawn under each peak to isolate intensity associated with the test line. The area under the peak was then quantified using the Wand tool. Values were subsequently plotted in Prism. Cas13a detection (top) and Cas12a detection (bottom).

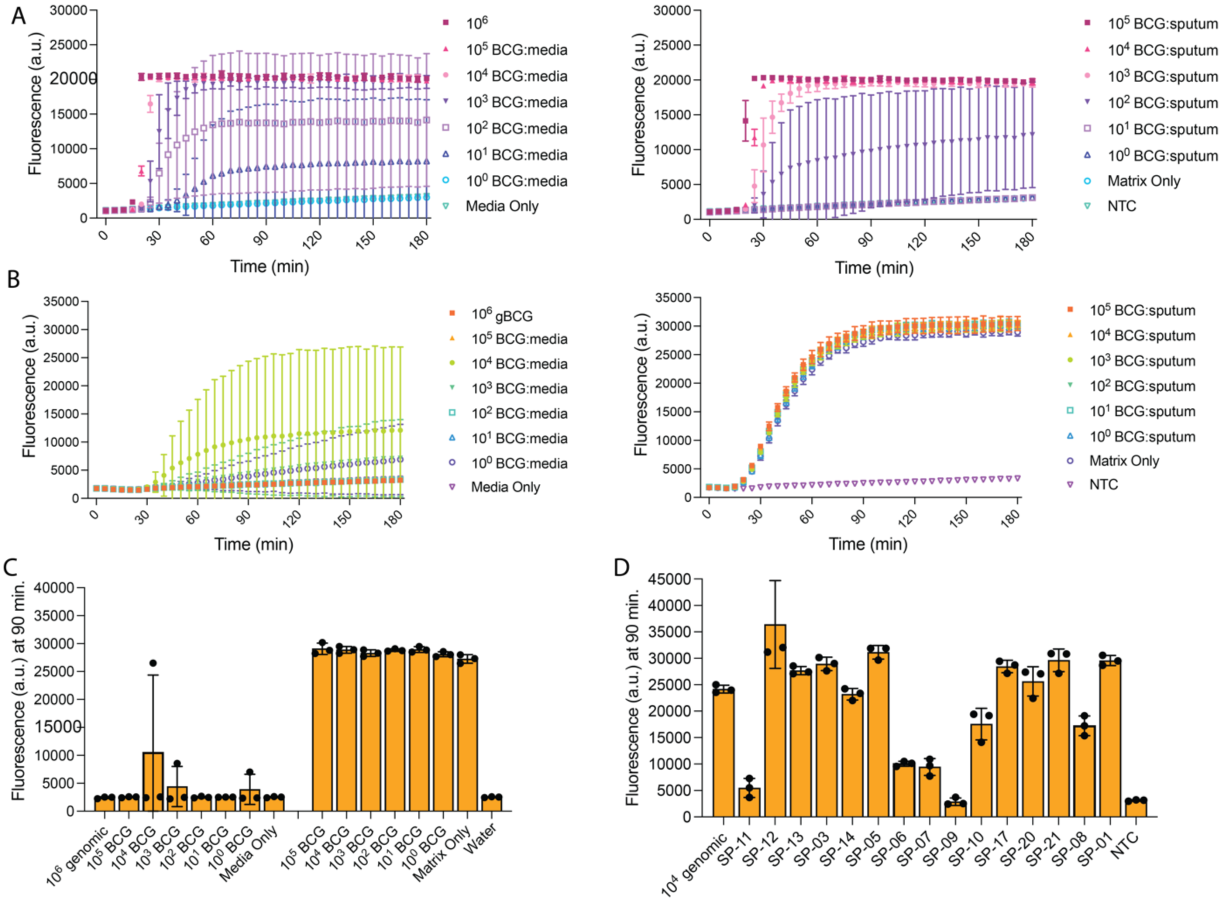

Fig. S9. Internal control and fluorescence kinetics data for Fig. 3D and 3E. A. Fluorescence kinetic plots for Fig. 3D, as reported by the Cas13a target fluorophore (FAM). **B.** Fluorescence kinetic plots for Fig. 3D, as reported by Cas12a target fluorophore (HEX). **C.** Cas12a internal control fluorescence from Fig. 3D. **D.** Cas12a internal control fluorescence from Fig. 3E. In all panels, error bars: SD based on n=3 technical replicates.

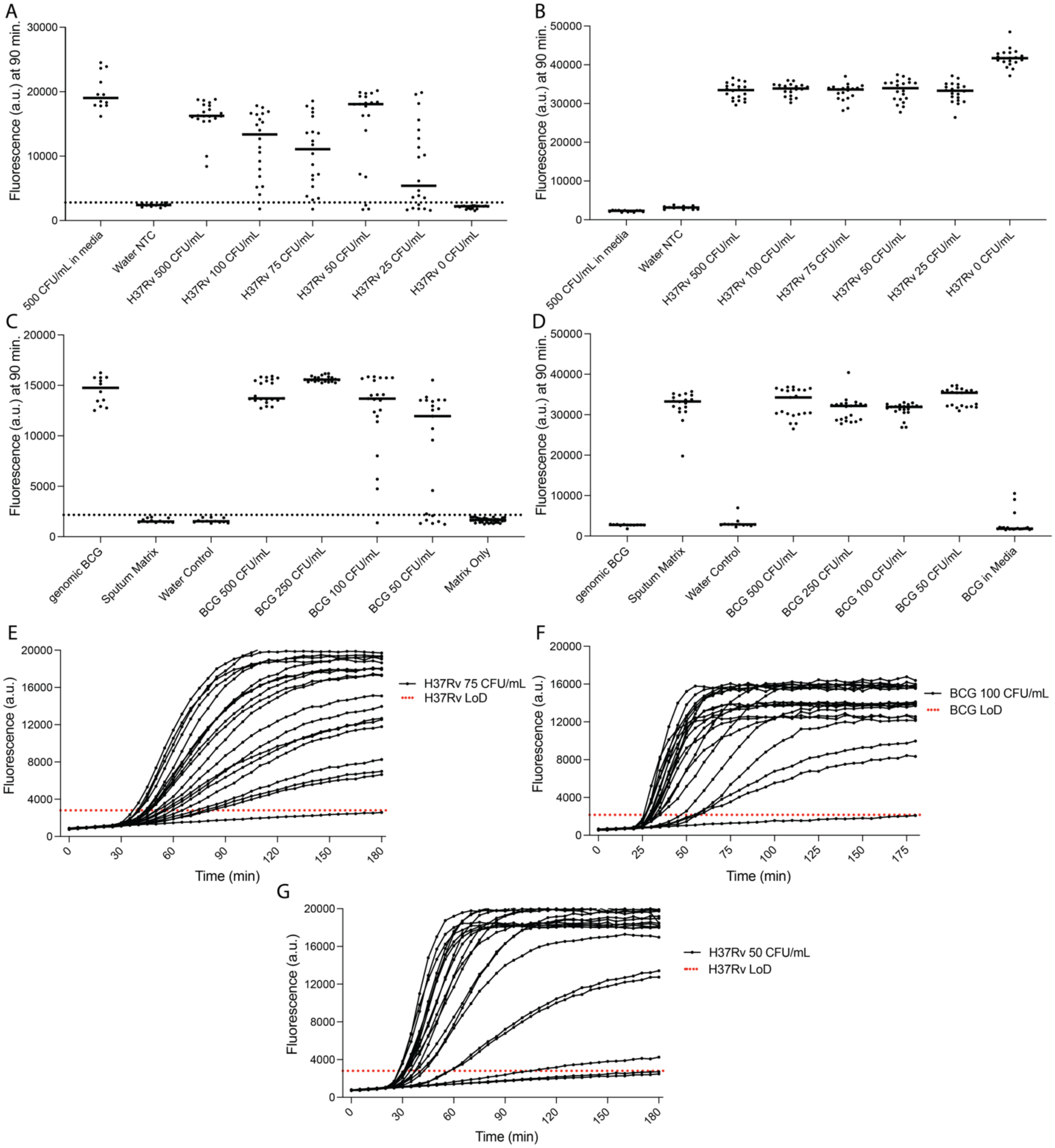

Figure S10. Additional data for Fig. 3 LoD determination. A. Individual fluorescence values from Fig. 3F, H37Rv LoD. Cas13a *Mtb*. **B.** Individual fluorescence values from Fig. 3F, H37Rv LoD. Cas12a internal control. **C.** Individual fluorescence values from Fig. 3G, BCG LoD. Cas13a *Mtb*. **D.** Individual fluorescence values from Fig. 3G, BCG LoD. Cas12a internal control. **E.** Fluorescence kinetics from Fig. 3F, H37Rv LoD. Cas13a *Mtb* detecting 100 CFU/mL. **F.** Fluorescence kinetics from Fig. 3G, BCG LoD. Cas13a *Mtb* detecting 75 CFU/mL. **G.** Fluorescence kinetics for Cas13a detecting 50 CFU/mL of H37Rv. All units are in CFU/mL. All Cas13a assays are dual-detection (IS6110 C and IS1081 A).

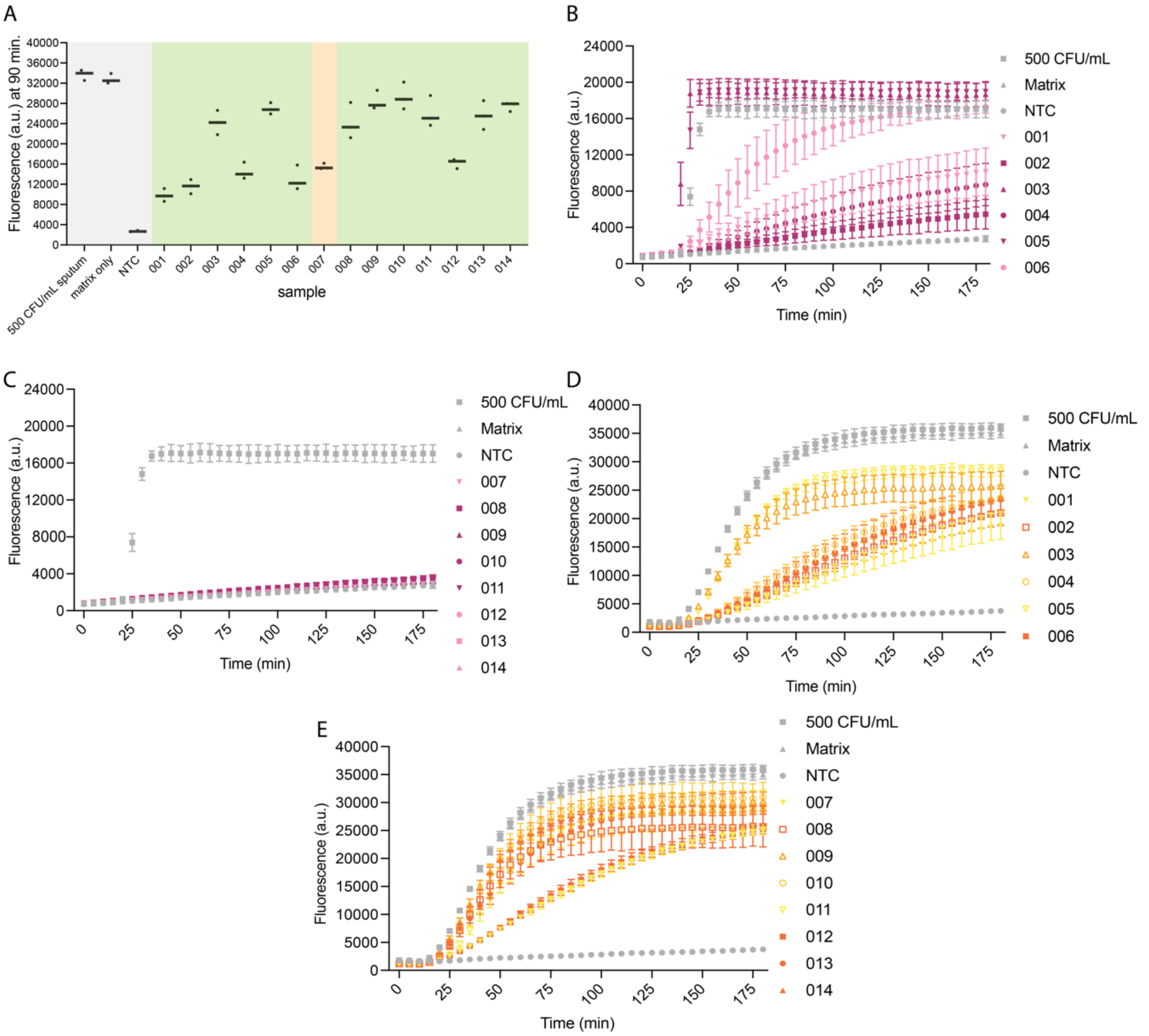

Figure S11. Internal control and fluorescence kinetics for Fig. 4. A. Internal control data for Fig. 4B. **B.** Cas13a fluorescence kinetics data for positive samples seen in Fig. 4B. **C.** Cas13a fluorescence kinetics data for negative samples seen in Fig. 4B. **D.** Internal control data for positive samples seen in Fig. 4B. **E.** Internal control data for negative samples seen in Fig. 4B. In **B-E**, error bars: SD based on n=3 technical replicates.

Table S1. Comparison of SHINE-TB to existing CRISPR-TB methods.

Ref.	Sample to answer time (min)	Combined amp + detection	Readout	Equipment required[a]	Compatible with lyophilization	Analytical sensitivity (cp/uL)[b]	Contrived sample LoD (CFU/mL)	Clinical Sensitivity	Clinical Specificity	Multi-target	Cas Protein(s)	Compatible sample type(s)
**Respiratory samples**												
TB-One Pot (27)	80	*Yes*	Fluorescence	RT-PCR system or UV transilluminator, centrifuge, vortexer, and automated nucleic acid extraction system	*N.D.*	0.8[c]	50[c]	67%	97%	*No*	AapCas12b	Sputum
MTB-MCDA-CRISPR (34)	N.D.[d]	*No*	Fluorescence	RT-PCR system or blue-light transilluminator[e]	*N.D.*	~8[f]	N.D.[g]	100%	100%	*No*	LbaCas12a	Sputum
TB-QUICK (30)	~135[h]	*No*	Fluorescence	RT-PCR system, centrifuge, heat block/water bath, and vortexer[i]	*N.D.*	1.3[c]	N.D.	87%	95%	*No*	AacCas12b	Sputum, others[j]
CRISPR-MTB (28)	~110[k]	*No*	Fluorescence	RT-PCR system, heat block or water bath, centrifuge, and vortexer	*N.D.*	5[l]	50[1]	79%	98%	*No*	LbaCas12a	Sputum, others[j]
LACD (29)	~60[m]	*No*	Fluorescence or Lateral Flow	RT-PCR system[n], water bath, centrifuge	*N.D.*	~10[o]	N.D.	80%	N.D.	*No*	LbaCas12a	Sputum
(37)	N.D.	*No*	Fluorescence or naked eye	RT-PCR system[n] or UV spectrophotometer[n], and heat block[c]	*N.D.*	1[p]	N.D.	100%	100%	*No*	LbaCas12a	Sputum
CRISPR-Live-MTB (36)	~175-235[q]	*No*	Fluorescence or Lateral Flow	Microplate reader[n], sonicator, water bath, vortexer, and centrifuge	*N.D.*	0.6[r]	N.D.	95%	100%	*No*	LwaCas13a	Sputum, BALF puncture
(31)	~60[s]	*No*	Fluorescence or Lateral Flow	Microplate reader[n], heat block	*N.D.*	1[t]	N.D.	80%	100%	*No*	Lba Cas12a	BALF, others[j]
CRISPR-MCDA (35)	~70[u]	*No*	Fluorescence	RT-PCR system[v] or UV transilluminator, heat block, centrifuge	*N.D.*	~1[w]	N.D.	80.95%	100%	*No*	AapCas12b	Sputum
CRISPR-MTB (73)	~120[x]	*No*	Fluorescence	RT-PCR system, Vortexer, centrifuge, thermocycler	*N.D.*	1[y]	N.D.	97.4%	95.5%	*No*	LwCas13a	BALF, Sputum, Pus

(74)	240[z]	*No*	Fluorescence	RT-PCR system, BioRobot MDx or the BioRobot Universal System[aa], centrifuge, heat block	*N.D.*	2700 [bb]	N.D.	99.29%	100%	*No*	LbCas12a	Sputum
**This study SHINE-TB**	~125[cc]	*Yes*	Fluorescence or Lateral Flow	Microplate reader[n], heat block, vortexer, and low-speed centrifuge	*Yes*	1-4.5[dd]	69 - 80.5[ee]	100%	100%	*Yes*	LwaCas13a LbaCas12a	Sputum
**Other sample types (blood, plasma, cfDNA)**												
WATSON (33)	220[ff]	*No*	Fluorescence or Lateral Flow	Microplate reader[n], low-speed centrifuge, Maxwell RSC system (Promega)	*N.D.*	0.01–0.1[gg]	N.D.	78-91%[hh]	100%	*Yes*	LwaCas13a	plasma
CRISPR-TB (32)	120[ii]	*No*	Fluorescence or Lateral Flow	Thermocycler and microplate reader[n], water bath or heat block, microcentrifuge , vacuum manifold or centrifuge	*N.D.*	0.06[jj]	N.D.	96%[kk]	94%[kk]	*No*	LbaCas12a	serum
**TB-One Pot** (38)	~80[ll]	*Yes*	Fluorescence	RT-PCR system, centrifuge, vortexer, heat block	*N.D.*	N.D.	N.D.	65%	96.30%	*No*	AapCas12b	pleural effusion samples (cell-free Mtb)
(75)	N.D.	*N/A*[mm]	Electrochemical	VSP potentiostat[nn]	*N.D.*	$> 10^9$	N.D.	N.D.	N.D.	*No*	LbaCas12a	N/A

[a] Equipment required lists all equipment from sample processing to readout. Equipment required by extraction kits included. Low-speed centrifuge applies to papers that explicitly indicate rotation forces below 10,000 rcf.
[b] Background is assumed to be water unless otherwise noted by the paper.
[c] Input: H37Rv; replicates: 10.
[d] Not Determined for sample to answer time. N.D. refers to timing that was either not described in the text or when sample extraction method/timing was missing/inadequately described to determine sample-to-answer time.
[e] Equipment needed for extraction not reported.
[f] Input: H37Rv; replicates: 3; 40 fg per reaction reported.
[g] N.D. for sensitivity and specificity refers to assays that were not performed, reported, or thoroughly described.
[h] Calculated: 45 min extraction, 80 min LAMP for sputum, 10 min detection. Reported as under 2 hours.
[i] Assuming centrifuge-based protocol from extraction kits. See paper for details.
[j] For other sample types, see paper.
[k] Calculated: 1 hour extraction, 30 min RPA, 20 min detection. Paper reports extraction at 40 min.
[l] Input: H37Ra MTBs; background: negative sputum; replicates: 10.
[m] As reported by paper: 15 min template extraction, 35-40 min LAMP reaction, 5 min detection.
[n] Only required for fluorescence measurements.
[o] Calculated based on 50 fg genomic DNA per reaction with 1 µL of input. Input: H37Rv; replicates: 3.
[p] Input: *Mtb* DNA (not described); replicates: unknown
[q] Calculated: 40 min extraction, 72-132 min amplification, 60 min detection.
[r] Calculated: 2.4 copies reported, assuming this is total copies added in 4 µL of sample, corresponding to 0.2 aM template concentration. Input: *Mtb* RNA (H37Rv); replicates: 3.
[s] Calculated: 10-15 min extraction (for clinical samples), 20 min amplification, 20 min detection.
[t] Input: H37Rv; replicates: 3.
[u] As reported by paper: 25 min extraction, 40 min amplification, 5 min detection. 30 min needed for visual detection under UV.
[v] Exact equipment not specified.
[w] Calculated based on 5 fg/µL. Input: H37Rv; replicates: 20.
[x] Calculated: 60 min extraction, ~30 min amplification, 30 min detection.
[y] Input: IS1081 plasmid; replicates: 3.
[z] As reported by paper: 60 min extraction, 60 min amplification, 120 min detection.
[aa] Based on user manual for extraction kit.
[bb] Calculated from 4.48 fmol/L. Input: IS1081 plasmid; replicates: 3.
[cc] ~35 min extraction, 90 min combined amplification/detection reaction. Add ~5 min if doing lateral flow.
[dd] Input: BCG; background: diluted negative sputum; replicates: 20 copies. Based on dPCR supplemental table 3.
[ee] Input: H37Rv and BCG, respectively; background: diluted negative sputum; replicates: 20 or 21.
[ff] Calculated: 70 min extraction, 30 min amplification, 120 min detection.
[gg] Calculated: assuming a 50 µL reaction and 1/50 of volume was DNA template. Input: H37Rv *Mtb* gDNA fragmented; background: gDNA in water; replicates: 6 replicates.
[hh] Corresponding to small input volume (4 µL) and large input volume (400 µL), respectively.
[ii] Excluding time required for serum isolation.
[jj] Input: serum cfDNA concentrated; replicates: 3.
[kk] For adult samples tested.
[ll] Calculated: >45 min extraction based on TIANamp Magnetic DNA Kit user manual, ~32 min reaction time.
[mm] Amplification-free method.
[nn] No extracted method/extracted samples reported.

Table S2. dPCR data on sputum dilutions for Dynamic Range experiment 3D.

(Sputum+BCG):TE IS6110 copies/µL						
**CFU/ml**	**rep 1**		**rep 2**		**rep 3**	
**10^5**	844.8	834.6	1048.8	1111.2	652.2	636.6
**10^4**	100.2	66.12	55.572	74.7	155.28	143.52
**10^3**	11.334	18.522	14.196	9.348	14.292	14.496
**10^2**	4.548	0	9.432	N/A	4.764	0
**10^1**	0	0	0	2.382	0	0
**10^0**	0	0	0	0	0	0
**0**	0	0	0	0	0	0
**NTC**	0	0				

Table S3. dPCR data on media dilutions for Dynamic Range experiment 3D.

(media+BCG):TE	IS6110 copies/µL					
**CFU/ml**	**rep 1**		**rep 2**		**rep 3**	
**10^5**	1881.6	1873.2	2004	2146.2	1392	1381.2
**10^4**	249.18	264.72	119.34	112.68	81.42	129.48
**10^3**	14.37	7.182	18.708	N/A	23.028	15.978
**10^2**	0	2.406	2.424	0	5.028	0
**10^1**	0	0	0	2.4	0	0
**10^0**	0	0	0	0	0	0
**0**	0	0	0	0	0	0
**NTC**	0	0				

Table S4. dPCR results for BCG LoD.

CFU/mL	Sputum:TE IS6110 copy # per µL	Media:TE IS6110 copy # per µL	Control
1000	27.2	33.46	NA
750	28.44	43.12	NA
500	12.78	13.16	NA
250	9.64	11.1	NA
100	3.22	4.42	NA
50	1.08	1.1	NA
Negative Control	0	0	NA
H37Ra gDNA 10^3 copies	NA	NA	44238
NTC	NA	NA	0

Table S5. Patient sample information for Fig. 4.

#	Patient #	Age and Gender	Xpert (Colombia)	Xpert Ct Value (Colombia) IS-1081/6110	dPCR copies/2µL (Rutgers) IS-1081/6110	Description
001	TB-02-0019	41, M	Medium	16.2	10890.06667	Smear (+), Culture (+), GeneXpert sputum (+)
002	TB-01-0083	24, M	High	15.9	16065.7	Smear (+), Culture (+), GeneXpert sputum (+)
003	TB-01-0111	47, M	Medium	15.8	358.6633333	Smear (+), Culture (+), GeneXpert sputum (+)
004	TB-01-0112	59, M	High	15.9	37198	Smear (+), Culture (+), GeneXpert sputum (+)
005	TB-01-0120	35, M	Low	16.5	34	Smear (+), Culture (+), GeneXpert sputum (+)
006	TB-01-0159	53, F	Medium	16	3453.76	Smear (+), Culture (+), GeneXpert sputum (+)
007	TB-01-0162	23, M	Low	20.2	0	Smear (ND), Culture (-), GeneXpert sputum (+)
008	TB-02-0018	37, F	Not Detected	0	NA	Smear (-), Culture (C), GeneXpert sputum (-)
009	TB-02-0020	30, M	Not Detected	0	NA	Smear (-), Culture (-), GeneXpert sputum (-)

010	TB-02-0021	61, M	Not Detected	0	NA	Smear (-), Culture (-), GeneXpert sputum (-)
011	TB-02-0022	32, F	Not Detected	0	NA	Smear (-), Culture (-), GeneXpert sputum (-)
012	TB-02-0023	68, M	Not Detected	0	NA	Smear (ND), Culture (-), GeneXpertsputum (-)
013	TB-01-0137	59, M	Not Detected	0	NA	Smear (-), Culture (-), GeneXpert sputum (-)
014	TB-01-0145	70, F	Not Detected	0	NA	Smear (-), Culture (-), GeneXpert sputum (-)

Table S6. Fluorescence cutoff point derivation and associated sensitivities and specificities.

	Cutoff Value (FU)	Sensitivity (95% CI)	Specificity (95% CI)
**Mean + 3 SD of negative controls**			
Training set (n = 83)	3287.03	0.93 (0.85, 1.00)	1.00 (1.00, 1.00)
Test set (n = 32)		1.00 (1.00, 1.00)	1.00 (1.00, 1.00)
**Youden index**			
Training set (n = 83)	2895.42	0.96 (0.89, 1.00)	1.00 (1.00, 1.00)
Test set (n = 32)		0.93 (0.80, 1.00)	1.00 (1.00, 1.00)
**TPP 2024 sensitivity standard***			
Training set (n = 83)	7047.50	0.85	1.00 (1.00, 1.00)
Test set (n = 32)		0.82 (0.65, 1.00)	1.00 (1.00, 1.00)
**TPP 2024 specificity standard**			
Training set (n = 83)	2895.42	0.96 (0.89, 1.00)	0.98
Test set (n = 32)		0.93 (0.80, 1.00)	1.00 (1.00, 1.00)

*For a sputum-based near-POC test.

Table S7. Gene Block Fragments Ordered.

Name		Oligo type; company	Sequence	Relevant Figures
IS6110_A_target	Synthetic Fragment	gBlocks gene fragments; IDT	GAACCGGATCGATGTGTACTGAGATCCCCTATCCGTATGGTGGATAACGTCTTTCAGGTCGAGTACGCCTTCTTGTTGGCGGGTCCAGATGGCTTGCTCGATCGCGTCGAGGACCATGGAGGTGGCCATCGTGGAAGCGACCCGCCAGCCCAGGATCCTGCGAGCGTAGGCGTCGGTGACAAAGGCCACGTAGGCGAACCCTGCCCAGGTCGACACATAGGTGAGGTCTGCTACCCACAGCCGGTTAGGTGCTGGTGGTCCGAAGCGGCGCTGGACGAGATCGGCGGGACGGGCTGTGGCCGGATCAGCGATCGTGGTCCTGCGGGCTTTGCCGCGGGTGGTCCCGGACAGGCCGAGTTTGGTCATCAGCCGTTCGACGGTGCATCTGGCCACCTCGATGCCCTCACGGTTCAGGGTTAGCCACACTTTGCGGGCACCGTAAACACCGTAGTTGGCGGCGTGGACGCGGCTGATGTGCTCCTTGAGTTCGCCATCGCGCAGCTCGCGGCGGCTGGGCTCCCGGTTGATGTGGTCGTAGTAGGTCGATGGGGCGATCGGCACACCCAGCTCGGTCAGCTGTGTGCAGATCGACTCGACACCCCACCGCAAACCATCGGGGCCCTCGCGGTGGCCCTGATGATCGGCGATGAACCGGGTAATTAGCGTGCTGGCCGGTCGAGCTCGGCCGCGAAGAAAGCCGACGCGGTCTTTAAAATCGCGTTCGCCCTTCGCAATTCGGCGTTGTCCCGCCGCAAGCGCTTCAGCTCAGCGGATTCTTCGGTCGTGGTC	1 B, C and D; 2 A and B
IS6110_B_target	Synthetic Fragment	gBlocks gene fragments; IDT	AACCAGTCGACCCAGCGCGCGGTGGCCAACTCGACATCCTCGATGGACCGCCAGGGCTTGCCGGGTTTGATCAGCTCGGTCTTGTATAGGCCGTTGATCGTCTCGGCTAGTGCATTGTCATAGGAGCTTCCGACCGCTCCGACCGACGGTT	2 A
IS1081_A1_ta	Synthetic	gBlocks	GGAAGATGCCCACGACGTCGGTTC	1 B and D

rget	Fragment	gene fragments; IDT	GGCGTCGTACCTCTCGGTTGAGGCGTTCCTGGGGGTTGTTGGACCAGATTTGGCGCCAGATCTGCTTGGGGAAGGCGGTGAACGCCAGCAGGTCGGTGCGGGCGGTGTCGAGGTGCTCGGCCACCGCGGGGAGTTTGTCGGTCAGAGCGTCGAGTACCCGATCATATTGGGCAACAACTGATTCGGCGTCGGGCTGGTCGTAGATGGAGTGCAGCAGGGTGCGC	
IS1081_A2_target	Synthetic Fragment	gBlocks gene fragments; IDT	AGGTATACGGGCCGGCATCGAGCGGGCGGGTCCGAAACGCCTCTACGGCTTCGTCGAGCTCTTTGGCCATGATCGACACTTGCGACTTGGAAAGCTTTGTCACACCAAGTGTTTCGACCAGGCGCTCCATCCGGCGAGTGGATACTCCCAGCAGGTAGCAGGTCGCCACCACGCTGGTCAGTGCGCGTTCAGCTCGCTTGCGGCGCTGCAGCA	1 B and D; 2 A
IS1081_C,B_target	Synthetic Fragment	gBlocks gene fragments; IDT	TCCGGCGAGTGGATACTCCCAGCAGGTAGCAGGTCGCCACCACGCTGGTCAGTGCGCGTTCAGCTCGCTTGCGGCGCTGCAGCAGCCAGTCCGGGAAATAGCTGCCCTGGCGCAGCTTGGGGATCGCGACGTCGATGGTTGCGGCACGGGTGTCGAAATCACGGTGGCGGTAGCCGTTGCGCTGATTGGACCGCTCATCGCTGCGTTCGCGGTAGCCCGCCCCGCACAGGGCGTCGGCTTCAGCCCCCATCAAGGCGGCGATGAACGTCGAGAGCAGCCCGC	1 B, C, and D; 2 A
LR5_target_3	Synthetic Fragment	gBlocks gene fragments; IDT	ggtgggacatgcgggcagcaatactgctttgtaaagcattgagatgtttatgtgtatgcatatctaaaagcacagcacttaatcctttaccttgtctatgatgcaaagacctttgttcacgtgtttgtctgctga	3 B; Supplemental 4 E
LR5_target_1-2	Synthetic Fragment	gBlocks gene fragments; IDT	cccgtgctctctgaaacatgtgctgtgtcaactcagggttaaatggattaagggcggtgcaagatgtgctttgttaaacagatgcttgaaggcagcatgctccttaagagtcatcaccactccctaatctcaagtacccagggacacaaaaactgcggaagg	Supplemental 4 C and D

Table S8. Primer, crRNA, and reporter sequences.

Name	Label	Oligo type; company	Sequence	Gene assay name
IS6110_cr1	crRNA	custom RNA; IDT	/AltR1/rUrArArUrUrUrCrUrArCrUrArArGrUrGrUrArGrArUrCrUrArCrCrCrArCrArGrCrCrGrGrUrUrArGrGrU/AltR2/	Cas12_IS6110_cr1
IS6110_cr2	crRNA	custom RNA; IDT	/AltR1/rUrArArUrUrUrCrUrArCrUrArArGrUrGrUrArGrArUrArCrCrGrGrCrUrGrUrGrGrGrUrArGrCrArGrArC/AltR2/	Cas12_IS6110_cr2
IS6110_cr3	crRNA	custom RNA; IDT	/AltR1/rUrArArUrUrUrCrUrArCrUrArArGrUrGrUrArGrArUrArArArGrArCrCrGrCrGrUrCrGrGrCrUrUrUrCrU/AltR2/	Cas12_IS6110_cr3
IS6110_cr4	crRNA	custom RNA; IDT	/AltR1/rUrArArUrUrUrCrUrArCrUrArArGrUrGrUrArGrArUrCrCrGrCrGrGrGrUrGrGrUrCrCrCrGrGrArCrArG/AltR2/	Cas12_IS6110_cr4
IS6110_cr5	crRNA	custom RNA; IDT	/AltR1/rUrArArUrUrUrCrUrArCrUrArArGrUrGrUrArGrArUrCrGrGrGrCrArCrCrGrUrArArArCrArCrCrGrUrA/AltR2/	Cas12_IS6110_cr5
IS6110_cr6	crRNA	custom RNA; IDT	/AltR1/rUrArArUrUrUrCrUrArCrUrArArGrUrGrUrArGrArUrArArArUrCrGrCrGrUrUrCrGrCrCrCrUrUrCrGrC/AltR2/	Cas12_IS6110_cr6
IS1081_cr1	crRNA	custom RNA; IDT	/AltR1/rUrArArUrUrUrCrUrArCrUrArArGrUrGrUrArGrArUrGrCrGrCrCrArGrArUrCrUrGrCrUrUrGrGrGrGrA/AltR2/	Cas12_IS1081_cr1
IS1081_cr3	crRNA	custom RNA; IDT	/AltR1/rUrArArUrUrUrCrUrArCrUrArArGrUrGrUrArGrArUrGrCrCrArUrGrArUrCrGrArCrArCrUrUrGrCrGrA/AltR2/	Cas12_IS1081_cr3
IS1081_cr4	crRNA	custom RNA; IDT	/AltR1/rUrArArUrUrUrCrUrArCrUrArArGrUrGrUrArGrArUrUrCrArCrArCrCrArArGrUrGrUrUrUrCrGrArCrC/AltR2/	Cas12_IS1081_cr4
C13_IS6110_A	crRNA	custom RNA; IDT	/AltR1/rGrArUrUrUrArGrArCrUrArCrCrCrCrArArArArArCrGrArArGrGrGrGrArCrUrArArArArCrGrGrCrArUrCrGrArGrGrUrGrGrCrCrArGrArUrGrCrArCrCrGrUrCrG/AltR2/	Cas13_IS6110_A
C13_IS6110_B	crRNA	custom RNA; IDT	/AltR1/rGrArUrUrUrArGrArCrUrArCrCrCrCrArArArArArCrGrArArGrGrGrGrArCrUrArArArArCrGrArCrGrArUrCrArArCrGrGrCrCrUrArUrArCrArArGrArCrCrGrArG/AltR2/	Cas13_IS6110_B
C13_IS6110_	crRNA	custom	/AltR1/rGrArUrUrUrArGrArCrUrArCrC	Cas13_IS6110

sg5		RNA; IDT	rCrCrArArArArArCrGrArArGrGrGrArCrUrArArArArCrCrUrArCrGrGrUrGrUrUrUrArCrGrGrUrGrCrCrCrGrCrArArArGrUrG/AltR2/	_C
C13_IS1081_A	crRNA	custom RNA; IDT	/AltR1/rGrArUrUrUrArGrArCrUrArCrCrCrCrArArArArArCrGrArArGrGrGrArCrUrArArArArCrCrArArArGrCrUrUrUrCrCrArArGrUrCrGrCrArArGrUrGrUrCrGrArU/AltR2/	Cas13_IS1081_A
C13_IS1081_B	crRNA	custom RNA; IDT	/AltR1/rGrArUrUrUrArGrArCrUrArCrCrCrCrArArArArArCrGrArArGrGrGrArCrUrArArArArCrGrCrArGrCrGrCrCrGrCrArArGrCrGrArGrCrUrGrArArCrGrCrGrCrA/AltR2/	Cas13_IS1081_B
C13_IS1081_C	crRNA	custom RNA; IDT	/AltR1/rGrArUrUrUrArGrArCrUrArCrCrCrCrArArArArArCrGrArArGrGrGrArCrUrArArArArCrGrCrGrGrGrCrUrArCrCrGrCrGrArArCrGrCrArGrCrGrArUrGrArGrC/AltR2/	Cas13_IS1081_C
LR5_crRNA_1	crRNA	custom RNA; IDT	/AlTR1/rUrArArUrUrUrCrUrArCrUrArArGrUrGrUrArGrArUrArCrArArArGrCrArCrArUrCrUrUrGrCrArCrCrGrC/AlTR2/	Cas12_internalControl_1
LR5_crRNA_2	crRNA	custom RNA; IDT	/AlTR1/rUrArArUrUrUrCrUrArCrUrArArGrUrGrUrArGrArUrArArGrGrArGrCrArUrGrCrUrGrCrCrUrUrCrArArG/AlTR2/	Cas12_internalControl_2
LR5_crRNA_3	crRNA	custom RNA; IDT	/AltR1/rUrArArUrUrUrCrUrArCrUrArArGrUrGrUrArGrArUrArArArGrCrArCrArGrCrArCrUrUrArArUrCrCrU/AltR2/	Cas12_internalControl_3
IS6110_cr1-2_F	Primer	custom DNA; IDT	CGTAGGCGAACCCTGCCCAGGTCGACACATAGG	Cas12_IS6110_cr1 & Cas12_IS6110_cr2
IS6110_cr1-2_R	Primer	custom DNA; IDT	CGATCTCGTCCAGCGCCGCTTCGGACCACCA	Cas12_IS6110_cr1 & Cas12_IS6110_cr2
IS6110_cr3_F	Primer	custom DNA; IDT	AATTAGCGTGCTGGCCGGTCGAGCTCGGCC	Cas12_IS6110_cr3
IS6110_cr3_R	Primer	custom DNA; IDT	GGGACAACGCCGAATTGCGAAGGGCGAACG	Cas12_IS6110_cr3

IS6110_cr4_F	Primer	custom DNA; IDT	TGTGGCCGGATCAGCGATCGTGGTCCTGCGGGCTT	Cas12_IS6110_cr4
IS6110_cr4_R	Primer	custom DNA; IDT	AGATGCACCGTCGAACGGCTGATGACCAAACTCGG	Cas12_IS6110_cr4
IS6110_cr5_F	Primer	custom DNA; IDT	TGGCCACCTCGATGCCCTCACGGTTCAGGGTTAGCCACAC	Cas12_IS6110_cr5
IS6110_cr5_R	Primer	custom DNA; IDT	GCGAACTCAAGGAGCACATCAGCCGCGTCCACGCCGCCAA	Cas12_IS6110_cr5 & Cas13_IS6110_C
IS6110_cr6_F	Primer	custom DNA; IDT	AGCTCGGCCGCGAAGAAAGCCGACGCGGTC	Cas12_IS6110_cr6
IS6110_cr6_R	Primer	custom DNA; IDT	TGAAGCGCTTGCGGCGGGACAACGCCGAAT	Cas12_IS6110_cr6
IS1081_cr1_F	Primer	custom DNA; IDT	ACCTCTCGGTTGAGGCGTTCCTGGGGGTTGTTGGACCAGA	Cas12_IS1081_cr1
IS1081_cr1_R	Primer	custom DNA; IDT	ACCTCGACACCGCCCGCACCGACCTGCTGGCGTTCACCGC	Cas12_IS1081_cr1
IS1081_cr3_F	Primer	custom DNA; IDT	GGTCCGAAACGCCTCTACGGCTTCGTCGAGCTCTT	Cas12_IS1081_cr3
IS1081_cr3_R	Primer	custom DNA; IDT	CCTGGTCGAAACACTTGGTGTGACAAAGCTTTCCA	Cas12_IS1081_cr3
IS1081_cr4_F	Primer	custom DNA; IDT	TTTGGCCATGATCGACACTTGCGACTTGGAAAGC	Cas12_IS1081_cr4
IS1081_cr4_R	Primer	custom DNA; IDT	ACCTGCTGGGAGTATCCACTCGCCGGATGGAGCGC	Cas12_IS1081_cr4
C13_FwT7_IS6_A	Primer	custom DNA; IDT	gaaatTAATACGACTCACTATAgggGTCCCGGACAGGCCGAGTTTGGTCATCAGCCGTT	Cas13_IS6110_A
C13_FwT7_IS6_B	Primer	custom DNA; IDT	gaaatTAATACGACTCACTATAgggTCGATGGACCGCCAGGGCTTGCCGGGTTTGATCA	Cas13_IS6110_B
C13_FwT7_IS6_sg5	Primer	custom DNA; IDT	gaaatTAATACGACTCACTATAgggCCACCTCGATGCCCTCACGGTTCAGGGTTAGCCA	Cas13_IS6110_C
C13_FwT7_IS1_A	Primer	custom DNA; IDT	gaaatTAATACGACTCACTATAgggCGCCTCTACGGCTTCGTCGAGCTCTTTGGCCATG	Cas13_IS1081_A
C13_FwT7_IS1_B	Primer	custom DNA; IDT	gaaatTAATACGACTCACTATAgggCCAGCAGGTAGCAGGTCGCCACCACGCTGGTCAG	Cas13_IS1081_B

C13_FwT7_IS1_C	Primer	custom DNA; IDT	gaaatTAATACGACTCACTATAgggTCACGGTGGCGGTAGCCGTTGCGCTGATTGGACC	Cas13_IS1081_C
C13_Rv_IS6_A	Primer	custom DNA; IDT	TTACGGTGCCCGCAAAGTGTGGCTAACCCTGAACCGTGAG	Cas13_IS6110_A
C13_Rv_IS6_B	Primer	custom DNA; IDT	TCGGAGCGGTCGGAAGCTCCTATGACAATGCACTAGCCGA	Cas13_IS6110_B
C13_Rv_IS6_sg5	Primer	Custom DNA; IDT	GCGAACTCAAGGAGCACATCAGCCGCGTCCACGCCGCCAA	Cas13_IS6110_C
C13_Rv_IS1_A	Primer	custom DNA; IDT	ACTCGCCGGATGGAGCGCCTGGTCGAAACACTTGGTGTGA	Cas13_IS1081_A
C13_Rv_IS1_B	Primer	custom DNA; IDT	CCAAGCTGCGCCAGGGCAGCTATTTCCCGGACTGGCTGCT	Cas13_IS1081_B
C13_Rv_IS1_C	Primer	custom DNA; IDT	TTCATCGCCGCCTTGATGGGGGCTGAAGCCGACGCCCTGT	Cas13_IS1081_C
LR5_1_FW	Primer	custom DNA; IDT	gaaacatgtgctgtgtcaactcagggttaaatggattaag	Cas12_internal Control_1
LR5_1_RV	Primer	custom DNA; IDT	tggtgatgactcttaaggagcatgctgccttcaagcatct	Cas12_internal Control_1
LR5_2_FW	Primer	custom DNA; IDT	tggattaagggcggtgcaagatgtgctttgttaaacagat	Cas12_internal Control_2
LR5_2_RV	Primer	custom DNA; IDT	tttgtgtccctgggtacttgagattagggagtggtgatga	Cas12_internal Control_2
LR5_3_FW	Primer	custom DNA; IDT	gggcagcaatactgctttgtaaagcattgagatgtttatg	Cas12_internal Control_3
LR5_3_RV	Primer	custom DNA; IDT	caaacacgtgaacaaaggtctttgcatcatagacaaggta	Cas12_internal Control_3
FAM-5C-Q	Reporter	custom DNA; IDT	/56-FAM/CCCCC/3IABkFQ/	Cas12 fluorescent assays
HEX-5C-Q	Reporter	custom DNA; IDT	/5HEX/CCCCC/3IABkFQ/	Cas12-13 dual fluorescence assays
FAM-6U-Q	Reporter	custom RNA; IDT	/56-FAM/rUrUrUrUrUrU/3IABkFQ/	Cas13 and Cas12-13 fluorescence assays
FAM-14U-Bio	Reporter	custom RNA; IDT	/56-FAM/rUrUrUrUrUrUrUrUrUrUrUrUrUrU/3Bio/	Cas13 LFAs

FAM-5C-Bio	Reporter	custom DNA; IDT	/56-FAM/CCCCC/3Bio/	Cas12 LFAs
FAM-5C-Dig	Reporter	custom DNA; IDT	/56-FAM/CCCCC/3Dig_N/	Cas12 LFAs
Bio-14C-FAM	Reporter	custom DNA; IDT	/5Biosg/CCCCCCCCCCCCCC/36-FAM/	Cas12 LFAs
FAM-14C-Dig	Reporter	custom DNA; IDT	/56-FAM/CCCCCCCCCCCCCC/3Dig_N/	Cas12 LFAs

REFERENCES AND NOTES

1. World Health Organization (WHO), "Global tuberculosis report 2024" (WHO, 2024).

2. A.-M. Demers, S. Verver, A. Boulle, R. Warren, P. van Helden, M. A. Behr, D. Coetzee, High yield of culture-based diagnosis in a TB-endemic setting. *BMC Infect. Dis.* **12**, 218 (2012).

3. K. R. Steingart, V. Ng, M. Henry, P. C. Hopewell, A. Ramsay, J. Cunningham, R. Urbanczik, M. D. Perkins, M. A. Aziz, M. Pai, Sputum processing methods to improve the sensitivity of smear microscopy for tuberculosis: A systematic review. *Lancet Infect. Dis.* **6**, 664–674 (2006).

4. H. E. Jenkins, A. W. Tolman, C. M. Yuen, J. B. Parr, S. Keshavjee, C. M. Pérez-Vélez, M. Pagano, M. C. Becerra, T. Cohen, Incidence of multidrug-resistant tuberculosis disease in children: Systematic review and global estimates. *Lancet* **383**, 1572–1579 (2014).

5. A. Kunkel, P. Abel Zur Wiesch, R. R. Nathavitharana, F. M. Marx, H. E. Jenkins, T. Cohen, Smear positivity in paediatric and adult tuberculosis: Systematic review and meta-analysis. *BMC Infect. Dis.* **16**, 282 (2016).

6. C. C. Boehme, M. P. Nicol, P. Nabeta, J. S. Michael, E. Gotuzzo, R. Tahirli, M. T. Gler, R. Blakemore, W. Worodria, C. Gray, L. Huang, T. Caceres, R. Mehdiyev, L. Raymond, A. Whitelaw, K. Sagadevan, H. Alexander, H. Albert, F. Cobelens, H. Cox, D. Alland, M. D. Perkins, Feasibility, diagnostic accuracy, and effectiveness of decentralised use of the Xpert MTB/RIF test for diagnosis of tuberculosis and multidrug resistance: A multicentre implementation study. *Lancet* **377**, 1495–1505 (2011).

7. E. Hsiang, K. M. Little, P. Haguma, C. F. Hanrahan, A. Katamba, A. Cattamanchi, J. L. Davis, A. Vassall, D. Dowdy, Higher cost of implementing Xpert® MTB/RIF in Ugandan peripheral settings: Implications for cost-effectiveness. *Int. J. Tuberc. Lung Dis.* **20**, 1212–1218 (2016).

8. S. G. Schumacher, W. A. Wells, M. P. Nicol, K. R. Steingart, G. Theron, S. E. Dorman, M. Pai, G. Churchyard, L. Scott, W. Stevens, P. Nabeta, D. Alland, K. Weyer, C. M. Denkinger, C.

Gilpin, Guidance for studies evaluating the accuracy of sputum-based tests to diagnose tuberculosis. *J Infect Dis* **220**, S99–S107 (2019).

9. A. East-Seletsky, M. R. O'Connell, S. C. Knight, D. Burstein, J. H. D. Cate, R. Tjian, J. A. Doudna, Two distinct RNase activities of CRISPR-C2c2 enable guide-RNA processing and RNA detection. *Nature* **538**, 270–273 (2016).

10. J. S. Gootenberg, O. O. Abudayyeh, J. W. Lee, P. Essletzbichler, A. J. Dy, J. Joung, V. Verdine, N. Donghia, N. M. Daringer, C. A. Freije, C. Myhrvold, R. P. Bhattacharyya, J. Livny, A. Regev, E. V. Koonin, D. T. Hung, P. C. Sabeti, J. J. Collins, F. Zhang, Nucleic acid detection with CRISPR-Cas13a/C2c2. *Science* **356**, 438–442 (2017).

11. C. Myhrvold, C. A. Freije, J. S. Gootenberg, O. O. Abudayyeh, H. C. Metsky, A. F. Durbin, M. J. Kellner, A. L. Tan, L. M. Paul, L. A. Parham, K. F. Garcia, K. G. Barnes, B. Chak, A. Mondini, M. L. Nogueira, S. Isern, S. F. Michael, I. Lorenzana, N. L. Yozwiak, B. L. MacInnis, I. Bosch, L. Gehrke, F. Zhang, P. C. Sabeti, Field-deployable viral diagnostics using CRISPR-Cas13. *Science* **360**, 444–448 (2018).

12. J. S. Chen, E. Ma, L. B. Harrington, M. Da Costa, X. Tian, J. M. Palefsky, J. A. Doudna, CRISPR-Cas12a target binding unleashes indiscriminate single-stranded DNase activity. *Science* **360**, 436–439 (2018).

13. S.-Y. Li, Q.-X. Cheng, J.-K. Liu, X.-Q. Nie, G.-P. Zhao, J. Wang, CRISPR-Cas12a has both cis- and trans-cleavage activities on single-stranded DNA. *Cell Res.* **28**, 491–493 (2018).

14. J. Arizti-Sanz, C. A. Freije, A. C. Stanton, B. A. Petros, C. K. Boehm, S. Siddiqui, B. M. Shaw, G. Adams, T.-S. F. Kosoko-Thoroddsen, M. E. Kemball, J. N. Uwanibe, F. V. Ajogbasile, P. E. Eromon, R. Gross, L. Wronka, K. Caviness, L. E. Hensley, N. H. Bergman, B. L. MacInnis, C. T. Happi, J. E. Lemieux, P. C. Sabeti, C. Myhrvold, Streamlined inactivation, amplification, and Cas13-based detection of SARS-CoV-2. *Nat. Commun.* **11**, 5921 (2020).

15. C. M. Ackerman, C. Myhrvold, S. G. Thakku, C. A. Freije, H. C. Metsky, D. K. Yang, S. H. Ye, C. K. Boehm, T.-S. F. Kosoko-Thoroddsen, J. Kehe, T. G. Nguyen, A. Carter, A. Kulesa, J. R. Barnes, V. G. Dugan, D. T. Hung, P. C. Blainey, P. C. Sabeti, Massively multiplexed nucleic acid detection with Cas13. *Nature* **582**, 277–282 (2020).

16. J. Arizti-Sanz, A. Bradley, Y. B. Zhang, C. K. Boehm, C. A. Freije, M. E. Grunberg, T.-S. F. Kosoko-Thoroddsen, N. L. Welch, P. P. Pillai, S. Mantena, G. Kim, J. N. Uwanibe, O. G. John, P. E. Eromon, G. Kocher, R. Gross, J. S. Lee, L. E. Hensley, B. L. MacInnis, J. Johnson, M. Springer, C. T. Happi, P. C. Sabeti, C. Myhrvold, Simplified Cas13-based assays for the fast identification of SARS-CoV-2 and its variants. *Nat. Biomed. Eng.* **6**, 932–943 (2022).

17. O. O. Abudayyeh, J. S. Gootenberg, S. Konermann, J. Joung, I. M. Slaymaker, D. B. T. Cox, S. Shmakov, K. S. Makarova, E. Semenova, L. Minakhin, K. Severinov, A. Regev, E. S. Lander, E. V. Koonin, F. Zhang, C2c2 is a single-component programmable RNA-guided RNA-targeting CRISPR effector. *Science* **353**, aaf5573 (2016).

18. A. Tambe, A. East-Seletsky, G. J. Knott, J. A. Doudna, M. R. O'Connell, RNA binding and HEPN-nuclease activation are decoupled in CRISPR-Cas13a. *Cell Rep.* **24**, 1025–1036 (2018).

19. J. S. Gootenberg, O. O. Abudayyeh, M. J. Kellner, J. Joung, J. J. Collins, F. Zhang, Multiplexed and portable nucleic acid detection platform with Cas13, Cas12a, and Csm6. *Science* **360**, 439–444 (2018).

20. E. A. Nalefski, N. Patel, P. J. Y. Leung, Z. Islam, R. M. Kooistra, I. Parikh, E. Marion, G. J. Knott, J. A. Doudna, A.-L. M. Le Ny, D. Madan, Kinetic analysis of Cas12a and Cas13a RNA-Guided nucleases for development of improved CRISPR-Based diagnostics. *iScience* **24**, 102996 (2021).

21. W. Feng, H. Peng, H. Zhang, M. Weinfeld, X. C. Le, A sensitive technique unravels the kinetics of activation and trans-cleavage of CRISPR-cas systems. *Angew. Chem. Int. Ed. Engl.* **63**, e202404069 (2024).

22. T. Iwamoto, T. Sonobe, K. Hayashi, Loop-mediated isothermal amplification for direct detection of *Mycobacterium tuberculosis* complex, *M. avium*, and *M. intracellulare* in sputum samples. *J. Clin. Microbiol.* **41**, 2616–2622 (2003).

23. D. S. Boyle, R. McNerney, H. Teng Low, B. T. Leader, A. C. Pérez-Osorio, J. C. Meyer, D. M. O'Sullivan, D. G. Brooks, O. Piepenburg, M. S. Forrest, Rapid detection of *Mycobacterium tuberculosis* by recombinase polymerase amplification. *PLOS ONE* **9**, e103091 (2014).

24. K. Nagai, N. Horita, M. Yamamoto, T. Tsukahara, H. Nagakura, K. Tashiro, Y. Shibata, H. Watanabe, K. Nakashima, R. Ushio, M. Ikeda, A. Narita, A. Kanai, T. Sato, T. Kaneko, Diagnostic test accuracy of loop-mediated isothermal amplification assay for Mycobacterium tuberculosis: Systematic review and meta-analysis. *Sci. Rep.* **6**, 39090 (2016).

25. N. Singpanomchai, Y. Akeda, K. Tomono, A. Tamaru, P. Santanirand, P. Ratthawongjirakul, Naked eye detection of the *Mycobacterium tuberculosis* complex by recombinase polymerase amplification-SYBR green I assays. *J. Clin. Lab. Anal.* **33**, e22655 (2019).

26. T. Dong, L. Qin, Z. Wang, C. Fan, C. Shen, P. Feng, Q. Kong, B. Ke, B. Ying, F. Li, Point-of-care diagnosis of tuberculosis using a portable nucleic acid test with distance-based readout. *Anal. Chem.* **96**, 20204–20212 (2024).

27. L. Peng, T. Fang, Q. Cai, H. Li, H. Li, H. Sun, M. Zhu, L. Dai, Y. Shao, L. Cai, Rapid detection of *Mycobacterium tuberculosis* in sputum using CRISPR-Cas12b combined with cross-priming amplification in a single reaction. *J. Clin. Microbiol.* **62**, e0092323 (2024).

28. J.-W. Ai, X. Zhou, T. Xu, M. Yang, Y. Chen, G.-Q. He, N. Pan, Y. Cai, Y. Li, X. Wang, H. Su, T. Wang, W. Zeng, W.-H. Zhang, CRISPR-based rapid and ultra-sensitive diagnostic test for *Mycobacterium tuberculosis*. *Emerg. Microbes Infect.* **8**, 1361–1369 (2019).

29. Y. Wang, J. Li, S. Li, X. Zhu, X. Wang, J. Huang, X. Yang, J. Tai, LAMP-CRISPR-Cas12-based diagnostic platform for detection of *Mycobacterium tuberculosis* complex using real-time fluorescence or lateral flow test. *Mikrochim. Acta* **188**, 347 (2021).

30. I. K. Sam, Y.-Y. Chen, J. Ma, S.-Y. Li, R.-Y. Ying, L.-X. Li, P. Ji, S.-J. Wang, J. Xu, Y.-J. Bao, G.-P. Zhao, H.-J. Zheng, J. Wang, W. Sha, Y. Wang, TB-QUICK: CRISPR-Cas12b-assisted rapid and sensitive detection of *Mycobacterium tuberculosis*. *J. Infect.* **83**, 54–60 (2021).

31. H. Li, X. Cui, L. Sun, X. Deng, S. Liu, X. Zou, B. Li, C. Wang, Y. Wang, Y. Liu, B. Lu, B. Cao, High concentration of Cas12a effector tolerates more mismatches on ssDNA. *FASEB J.* **35**, e21153 (2021).

32. Z. Huang, S. M. LaCourse, A. W. Kay, J. Stern, J. N. Escudero, B. M. Youngquist, W. Zheng, D. Vambe, M. Dlamini, G. Mtetwa, L. M. Cranmer, I. Njuguna, D. C. Wamalwa, E. Maleche-Obimbo, D. G. Catanzaro, C. J. Lyon, G. John-Stewart, A. DiNardo, A. M. Mandalakas, B. Ning, T. Y. Hu, CRISPR detection of circulating cell-free *Mycobacterium tuberculosis* DNA in adults and children, including children with HIV: A molecular diagnostics study. *Lancet Microbe* **3**, e482–e492 (2022).

33. S. G. Thakku, J. Lirette, K. Murugesan, J. Chen, G. Theron, N. Banaei, P. C. Blainey, J. Gomez, S. Y. Wong, D. T. Hung, Genome-wide tiled detection of circulating *Mycobacterium tuberculosis* cell-free DNA using Cas13. *Nat. Commun.* **14**, 1803 (2023).

34. N. Jia, C. Wang, X. Liu, X. Huang, F. Xiao, J. Fu, C. Sun, Z. Xu, G. Wang, J. Zhou, Y. Wang, A CRISPR-Cas12a-based platform for ultrasensitive rapid highly specific detection of *Mycobacterium tuberculosis* in clinical application. *Front. Cell. Infect. Microbiol.* **13**, 1192134 (2023).

35. X. Yang, J. Huang, Y. Chen, X. Ying, Q. Tan, X. Chen, X. Zeng, S. Lei, Y. Wang, S. Li, Development of CRISPR/Cas12b-based multiple cross displacement amplification technique for the detection of *Mycobacterium tuberculosis* complex in clinical settings. *Microbiol. Spectr.* **11**, e0347522 (2023).

36. Y. Wang, H. Lin, A. Yang, J. Huang, W. Ren, J. Dong, S. Wang, W. Xu, Y. Pang, J. Qu, J. Liu, Rapid and sensitive diagnosis of live *Mycobacterium tuberculosis* using clustered regularly interspaced short palindromic repeat-Cas13a point-of-care RNA testing. *View* **5**, 20230109 (2024).

37. L. Zhang, H. Bai, C. Zhang, X. He, J. Zou, W. Bai, J. Tang, W. Zhuang, J. Hu, Y. Yao, B. Ying, W. Hu, Dual-mode sensing of *Mycobacterium tuberculosis* with DNA-functionalized gold nanoparticles and asymmetric RPA-triggered PAM-free CRISPR system. *Sens. Actuators B Chem.* **424**, 136920 (2025).

38. L. Peng, T. Fang, L. Dai, L. Cai, Diagnostic value of cross-priming amplification combined with CRISPR-Cas12b in detecting cell-free DNA in tuberculous pleural effusion. *Open Forum Infect. Dis.* **11**, ofae674 (2024).

39. O. Piepenburg, C. H. Williams, D. L. Stemple, N. A. Armes, DNA detection using recombination proteins. *PLOS Biol.* **4**, e204 (2006).

40. N. Modi, O. R. S. Dunkley, A. Bell, E. Hennig, A. Wats, Y. Huang, N. Daivaa, C. A. Myhrvold, Y. L. Xie, P. P. Banada, Simplified co-extraction of total nucleic acids from respiratory samples for detection of *Mycobacterium tuberculosis* and SARS-CoV-2 optimized for compatibility across diagnostic platforms. medRxiv 25322880 [Preprint] (2025); https://doi.org/10.1101/2025.02.27.25322880.

41. M. Qiu, X.-M. Zhou, L. Liu, Improved strategies for CRISPR-Cas12-based nucleic acids detection. *J. Anal. Test.* **6**, 44–52 (2022).

42. S. Lu, X. Tong, Y. Han, K. Zhang, Y. Zhang, Q. Chen, J. Duan, X. Lei, M. Huang, Y. Qiu, D.-Y. Zhang, X. Zhou, Y. Zhang, H. Yin, Fast and sensitive detection of SARS-CoV-2 RNA using suboptimal protospacer adjacent motifs for Cas12a. *Nat. Biomed. Eng.* **6**, 286–297 (2022).

43. P. Chitale, A. D. Lemenze, E. C. Fogarty, A. Shah, C. Grady, A. R. Odom-Mabey, W. E. Johnson, J. H. Yang, A. M. Eren, R. Brosch, P. Kumar, D. Alland, A comprehensive update to the *Mycobacterium tuberculosis* H37Rv reference genome. *Nat. Commun.* **13**, 7068 (2022).

44. H. C. Metsky, N. L. Welch, P. P. Pillai, N. J. Haradhvala, L. Rumker, S. Mantena, Y. B. Zhang, D. K. Yang, C. M. Ackerman, J. Weller, P. C. Blainey, C. Myhrvold, M. Mitzenmacher, P. C. Sabeti, Designing sensitive viral diagnostics with machine learning. *Nat. Biotechnol.* **40**, 1123–1131 (2022).

45. K. H. Lok, W. H. Benjamin Jr., M. E. Kimerling, V. Pruitt, M. Lathan, J. Razeq, N. Hooper, W. Cronin, N. E. Dunlap, Molecular differentiation of *Mycobacterium tuberculosis* strains without IS*6110* insertions. *Emerg. Infect. Dis.* **8**, 1310–1313 (2002).

46. D. van Soolingen, P. W. Hermans, P. E. de Haas, J. D. van Embden, Insertion element IS1081-associated restriction fragment length polymorphisms in Mycobacterium tuberculosis complex species: A reliable tool for recognizing Mycobacterium bovis BCG. *J. Clin. Microbiol.* **30**, 1772–1777 (1992).

47. E. S. Lander, L. M. Linton, B. Birren, C. Nusbaum, M. C. Zody, J. Baldwin, K. Devon, K. Dewar, M. Doyle, W. FitzHugh, R. Funke, D. Gage, K. Harris, A. Heaford, J. Howland, L. Kann, J. Lehoczky, R. LeVine, P. McEwan, K. McKernan, J. Meldrim, J. P. Mesirov, C. Miranda, W. Morris, J. Naylor, C. Raymond, M. Rosetti, R. Santos, A. Sheridan, C. Sougnez, Y. Stange-Thomann, N. Stojanovic, A. Subramanian, D. Wyman, J. Rogers, J. Sulston, R. Ainscough, S. Beck, D. Bentley, J. Burton, C. Clee, N. Carter, A. Coulson, R. Deadman, P. Deloukas, A. Dunham, I. Dunham, R. Durbin, L. French, D. Grafham, S. Gregory, T. Hubbard, S. Humphray, A. Hunt, M. Jones, C. Lloyd, A. McMurray, L. Matthews, S. Mercer, S. Milne, J. C. Mullikin, A. Mungall, R. Plumb, M. Ross, R. Shownkeen, S. Sims, R. H. Waterston, R. K. Wilson, L. W. Hillier, J. D. McPherson, M. A. Marra, E. R. Mardis, L. A. Fulton, A. T. Chinwalla, K. H. Pepin, W. R. Gish, S. L. Chissoe, M. C. Wendl, K. D. Delehaunty, T. L. Miner, A. Delehaunty, J. B. Kramer, L. L. Cook, R. S. Fulton, D. L. Johnson, P. J. Minx, S. W. Clifton, T. Hawkins, E. Branscomb, P. Predki, P. Richardson, S. Wenning, T. Slezak, N. Doggett, J. F. Cheng, A. Olsen, S. Lucas, C. Elkin, E. Uberbacher, M. Frazier, R. A. Gibbs, D. M. Muzny, S. E. Scherer, J. B. Bouck, E. J. Sodergren, K. C. Worley, C. M. Rives, J. H. Gorrell, M. L. Metzker, S. L. Naylor, R. S. Kucherlapati, D. L. Nelson, G. M. Weinstock, Y. Sakaki, A. Fujiyama, M. Hattori, T. Yada, A. Toyoda, T. Itoh, C. Kawagoe, H. Watanabe, Y. Totoki, T. Taylor, J. Weissenbach, R. Heilig, W. Saurin, F. Artiguenave, P. Brottier, T. Bruls, E. Pelletier, C. Robert, P. Wincker, D. R. Smith, L. Doucette-Stamm, M. Rubenfield, K. Weinstock, H. M. Lee, J. Dubois, A. Rosenthal, M. Platzer, G. Nyakatura, S. Taudien, A. Rump, H. Yang, J. Yu, J. Wang, G. Huang, J. Gu, L. Hood, L. Rowen, A. Madan, S. Qin, R. W. Davis, N. A. Federspiel, A. P. Abola, M. J. Proctor, R. M. Myers, J. Schmutz, M. Dickson, J. Grimwood, D. R. Cox, M. V. Olson, R. Kaul, C. Raymond, N. Shimizu, K. Kawasaki, S. Minoshima, G. A. Evans, M. Athanasiou, R. Schultz, B. A. Roe, F. Chen, H.

Pan, J. Ramser, H. Lehrach, R. Reinhardt, W. R. McCombie, M. de la Bastide, N. Dedhia, H. Blöcker, K. Hornischer, G. Nordsiek, R. Agarwala, L. Aravind, J. A. Bailey, A. Bateman, S. Batzoglou, E. Birney, P. Bork, D. G. Brown, C. B. Burge, L. Cerutti, H. C. Chen, D. Church, M. Clamp, R. R. Copley, T. Doerks, S. R. Eddy, E. E. Eichler, T. S. Furey, J. Galagan, J. G. Gilbert, C. Harmon, Y. Hayashizaki, D. Haussler, H. Hermjakob, K. Hokamp, W. Jang, L. S. Johnson, T. A. Jones, S. Kasif, A. Kaspryzk, S. Kennedy, W. J. Kent, P. Kitts, E. V. Koonin, I. Korf, D. Kulp, D. Lancet, T. M. Lowe, A. McLysaght, T. Mikkelsen, J. V. Moran, N. Mulder, V. J. Pollara, C. P. Ponting, G. Schuler, J. Schultz, G. Slater, A. F. Smit, E. Stupka, J. Szustakowki, D. Thierry-Mieg, J. Thierry-Mieg, L. Wagner, J. Wallis, R. Wheeler, A. Williams, Y. I. Wolf, K. H. Wolfe, S. P. Yang, R. F. Yeh, F. Collins, M. S. Guyer, J. Peterson, A. Felsenfeld, K. A. Wetterstrand, A. Patrinos, M. J. Morgan, P. de Jong, J. J. Catanese, K. Osoegawa, H. Shizuya, S. Choi, Y. J. Chen, J. Szustakowki, International Human Genome Sequencing Consortium, Initial sequencing and analysis of the human genome. *Nature* **409**, 860–921 (2001).

48. T. Notomi, H. Okayama, H. Masubuchi, T. Yonekawa, K. Watanabe, N. Amino, T. Hase, Loop-mediated isothermal amplification of DNA. *Nucleic Acids Res.* **28**, E63 (2000).

49. C. C. Boehme, P. Nabeta, G. Henostroza, R. Raqib, Z. Rahim, M. Gerhardt, E. Sanga, M. Hoelscher, T. Notomi, T. Hase, M. D. Perkins, Operational feasibility of using loop-mediated isothermal amplification for diagnosis of pulmonary tuberculosis in microscopy centers of developing countries. *J. Clin. Microbiol.* **45**, 1936–1940 (2007).

50. D.-G. Wang, J. D. Brewster, M. Paul, P. M. Tomasula, Two methods for increased specificity and sensitivity in loop-mediated isothermal amplification. *Molecules* **20**, 6048–6059 (2015).

51. R. J. Meagher, A. Priye, Y. K. Light, C. Huang, E. Wang, Impact of primer dimers and self-amplifying hairpins on reverse transcription loop-mediated isothermal amplification detection of viral RNA. *Analyst* **143**, 1924–1933 (2018).

52. I. M. Lobato, C. K. O'Sullivan, Recombinase polymerase amplification: Basics, applications and recent advances. *Trends Analyt. Chem.* **98**, 19–35 (2018).

53. N. Singpanomchai, Y. Akeda, K. Tomono, A. Tamaru, P. Santanirand, P. Ratthawongjirakul, Rapid detection of multidrug-resistant tuberculosis based on allele-specific recombinase polymerase amplification and colorimetric detection. *PLOS ONE* **16**, e0253235 (2021).

54. D. S. Mota, J. M. Guimarães, A. M. D. Gandarilla, J. C. B. S. Filho, W. R. Brito, L. A. M. Mariúba, Recombinase polymerase amplification in the molecular diagnosis of microbiological targets and its applications. *Can. J. Microbiol.* **68**, 383–402 (2022).

55. D. A. Huyke, A. Ramachandran, V. I. Bashkirov, E. K. Kotseroglou, T. Kotseroglou, J. G. Santiago, Enzyme kinetics and detector sensitivity determine limits of detection of amplification-free CRISPR-Cas12 and CRISPR-Cas13 diagnostics. *Anal. Chem.* **94**, 9826–9834 (2022).

56. N. L. Welch, M. Zhu, C. Hua, J. Weller, M. E. Mirhashemi, T. G. Nguyen, S. Mantena, M. R. Bauer, B. M. Shaw, C. M. Ackerman, S. G. Thakku, M. W. Tse, J. Kehe, M.-M. Uwera, J. S. Eversley, D. A. Bielwaski, G. McGrath, J. Braidt, J. Johnson, F. Cerrato, G. K. Moreno, L. A. Krasilnikova, B. A. Petros, G. L. Gionet, E. King, R. C. Huard, S. K. Jalbert, M. L. Cleary, N. A. Fitzgerald, S. B. Gabriel, G. R. Gallagher, S. C. Smole, L. C. Madoff, C. M. Brown, M. W. Keller, M. M. Wilson, M. K. Kirby, J. R. Barnes, D. J. Park, K. J. Siddle, C. T. Happi, D. T. Hung, M. Springer, B. L. MacInnis, J. E. Lemieux, E. Rosenberg, J. A. Branda, P. C. Blainey, P. C. Sabeti, C. Myhrvold, Multiplexed CRISPR-based microfluidic platform for clinical testing of respiratory viruses and identification of SARS-CoV-2 variants. *Nat. Med.* **28**, 1083–1094 (2022).

57. S. T. Cole, R. Brosch, J. Parkhill, T. Garnier, C. Churcher, D. Harris, S. V. Gordon, K. Eiglmeier, S. Gas, C. E. Barry III, F. Tekaia, K. Badcock, D. Basham, D. Brown, T. Chillingworth, R. Connor, R. Davies, K. Devlin, T. Feltwell, S. Gentles, N. Hamlin, S. Holroyd, T. Hornsby, K. Jagels, A. Krogh, J. McLean, S. Moule, L. Murphy, K. Oliver, J. Osborne, M. A. Quail, M. A. Rajandream, J. Rogers, S. Rutter, K. Seeger, J. Skelton, R. Squares, S. Squares, J. E. Sulston, K. Taylor, S. Whitehead, B. G. Barrell, Deciphering the biology of Mycobacterium tuberculosis from the complete genome sequence. *Nature* **393**, 537–544 (1998).

58. M. Jing, R. Bond, L. J. Robertson, J. Moore, A. Kowalczyk, R. Price, W. Burns, M. A. Nesbit, J. McLaughlin, T. Moore, User experience analysis of AbC-19 Rapid Test via lateral flow immunoassays for self-administrated SARS-CoV-2 antibody testing. *Sci. Rep.* **11**, 14026 (2021).

59. D. Helb, M. Jones, E. Story, C. Boehme, E. Wallace, K. Ho, J. Kop, M. R. Owens, R. Rodgers, P. Banada, H. Safi, R. Blakemore, N. T. N. Lan, E. C. Jones-López, M. Levi, M. Burday, I. Ayakaka, R. D. Mugerwa, B. McMillan, E. Winn-Deen, L. Christel, P. Dailey, M. D. Perkins, D. H. Persing, D. Alland, Rapid detection of *Mycobacterium tuberculosis* and rifampin resistance by use of on-demand, near-patient technology. *J. Clin. Microbiol.* **48**, 229–237 (2010).

60. C. Schrader, A. Schielke, L. Ellerbroek, R. Johne, PCR inhibitors – Occurrence, properties and removal. *J. Appl. Microbiol.* **113**, 1014–1026 (2012).

61. S. Chakravorty, A. M. Simmons, M. Rowneki, H. Parmar, Y. Cao, J. Ryan, P. P. Banada, S. Deshpande, S. Shenai, A. Gall, J. Glass, B. Krieswirth, S. G. Schumacher, P. Nabeta, N. Tukvadze, C. Rodrigues, A. Skrahina, E. Tagliani, D. M. Cirillo, A. Davidow, C. M. Denkinger, D. Persing, R. Kwiatkowski, M. Jones, D. Alland, The new Xpert MTB/RIF ultra: Improving detection of *Mycobacterium tuberculosis* and resistance to rifampin in an assay suitable for point-of-care testing. *MBio* **8**, 10.1128/mbio.00812-17 (2017).

62. Y. Chang, M. Zhang, G. Liu, X. Wu, Q. Yan, C. Yang, L. Liu, Y. Feng, X. Xia, Rapid and sensitive detection of *Mycobacterium tuberculosis* using nested multi-enzyme isothermal rapid amplification in a single reaction. *Microbiol. Spectr.* **12**, e0088724 (2024).

63. World Health Organization (WHO), "Target product profiles for tuberculosis diagnosis and detection of drug resistance" (WHO, 2024).

64. M. S. Bhamla, B. Benson, C. Chai, G. Katsikis, A. Johri, M. Prakash, Hand-powered ultralow-cost paper centrifuge. *Nat. Biomed. Eng.* **1**, 0009 (2017).

65. V. Singh, K. Chibale, Strategies to combat multi-drug resistance in tuberculosis. *Acc. Chem. Res.* **54**, 2361–2376 (2021).

66. Z. Xu, D. Chen, T. Li, J. Yan, J. Zhu, T. He, R. Hu, Y. Li, Y. Yang, M. Liu, Microfluidic space coding for multiplexed nucleic acid detection via CRISPR-Cas12a and recombinase polymerase amplification. *Nat. Commun.* **13**, 6480 (2022).

67. M. P. Nicol, R. C. Wood, L. Workman, M. Prins, C. Whitman, Y. Ghebrekristos, S. Mbhele, A. Olson, L. E. Jones-Engel, H. J. Zar, G. A. Cangelosi, Microbiological diagnosis of pulmonary tuberculosis in children by oral swab polymerase chain reaction. *Sci. Rep.* **9**, 10789 (2019).

68. A. K. Luabeya, R. C. Wood, J. Shenje, E. Filander, C. Ontong, S. Mabwe, H. Africa, F. K. Nguyen, A. Olson, K. M. Weigel, L. Jones-Engel, M. Hatherill, G. A. Cangelosi, Noninvasive detection of tuberculosis by oral swab analysis. *J. Clin. Microbiol.* **57**, e01847-18 (2019).

69. P. Byanyima, S. Kaswabuli, E. Musisi, C. Nabakiibi, J. Zawedde, I. Sanyu, A. Sessolo, A. Andama, W. Worodria, L. Huang, J. L. Davis, Feasibility and sensitivity of saliva GeneXpert MTB/RIF ultra for tuberculosis diagnosis in adults in Uganda. *Microbiol. Spectr.* **10**, e0086022 (2022).

70. S. F. Altschul, W. Gish, W. Miller, E. W. Myers, D. J. Lipman, Basic local alignment search tool. *J. Mol. Biol.* **215**, 403–410 (1990).

71. F. Madeira, N. Madhusoodanan, J. Lee, A. Eusebi, A. Niewielska, A. R. N. Tivey, R. Lopez, S. Butcher, The EMBL-EBI Job Dispatcher sequence analysis tools framework in 2024. *Nucleic Acids Res.* **52**, W521–W525 (2024).

72. J. N. Zadeh, C. D. Steenberg, J. S. Bois, B. R. Wolfe, M. B. Pierce, A. R. Khan, R. M. Dirks, N. A. Pierce, NUPACK: Analysis and design of nucleic acid systems. *J. Comput. Chem.* **32**, 170–173 (2011).

73. W. Ren, Y. Zhou, H. Li, Y. Shang, X. Zhang, J. Yuan, S. Li, C. Li, Y. Pang, Development and clinical evaluation of a CRISPR/Cas13a-based diagnostic test to detect Mycobacterium tuberculosis in clinical specimens. *Front. Microbiol.* **14**, 1117085 (2023).

74. H. Xu, X. Zhang, Z. Cai, X. Dong, G. Chen, Z. Li, L. Qiu, L. He, B. Liang, X. Liu, J. Liu, An isothermal method for sensitive detection of Mycobacterium tuberculosis complex using

clustered regularly interspaced short palindromic repeats/Cas12a Cis and trans cleavage. *J. Mol. Diagn.* **22**, 1020–1029 (2020).

75. S. Taufiq, M. Nagatag, S. R. Abbas, K. Sode, An electrochemical biosensor for the detection of tuberculosis specific DNA with CRISPRCas12a and redox-probe modified oligonucleotide. *Heliyon* **10**, e40754 (2024).
